# Asymmetric cell division promotes therapeutic resistance in glioblastoma stem cells

**DOI:** 10.1172/jci.insight.130510

**Published:** 2021-02-08

**Authors:** Masahiro Hitomi, Anastasia P. Chumakova, Daniel J. Silver, Arnon M. Knudsen, W. Dean Pontius, Stephanie Murphy, Neha Anand, Bjarne W. Kristensen, Justin D. Lathia

**Affiliations:** 1 Cancer Impact Area, Lerner Research Institute, Cleveland Clinic, Cleveland, Ohio, USA.; 2 Department of Cardiovascular & Metabolic Sciences, Lerner Research Institute, Cleveland Clinic, Cleveland, Ohio, USA.; 3 Department of Molecular Medicine, Cleveland Clinic Lerner College of Medicine of Case Western Reserve University, Cleveland, Ohio, USA.; 4 Case Comprehensive Cancer Center, Case Western Reserve University, Cleveland, Ohio, USA.; 5 Department of Pathology, Odense University Hospital, Odense, Denmark.; 6 Department of Clinical Research, University of Southern Denmark, Odense, Denmark.; 7 Rose Ella Burkhardt Brain Tumor and Neuro-Oncology Center, Cleveland, Ohio, USA.

**Keywords:** Cell Biology, Stem cells, Brain cancer, Cancer

## Abstract

Asymmetric cell division (ACD) enables the maintenance of a stem cell population while simultaneously generating differentiated progeny. Cancer stem cells (CSCs) undergo multiple modes of cell division during tumor expansion and in response to therapy, yet the functional consequences of these division modes remain to be determined. Using a fluorescent reporter for cell surface receptor distribution during mitosis, we found that ACD generated a daughter cell with enhanced therapeutic resistance and increased coenrichment of EGFR and neurotrophin receptor (p75NTR) from a glioblastoma CSC. Stimulation of both receptors antagonized differentiation induction and promoted self-renewal capacity. p75NTR knockdown enhanced the therapeutic efficacy of EGFR inhibition, indicating that coinheritance of p75NTR and EGFR promotes resistance to EGFR inhibition through a redundant mechanism. These data demonstrate that ACD produces progeny with coenriched growth factor receptors, which contributes to the generation of a more therapeutically resistant CSC population.

## Graphical abstract

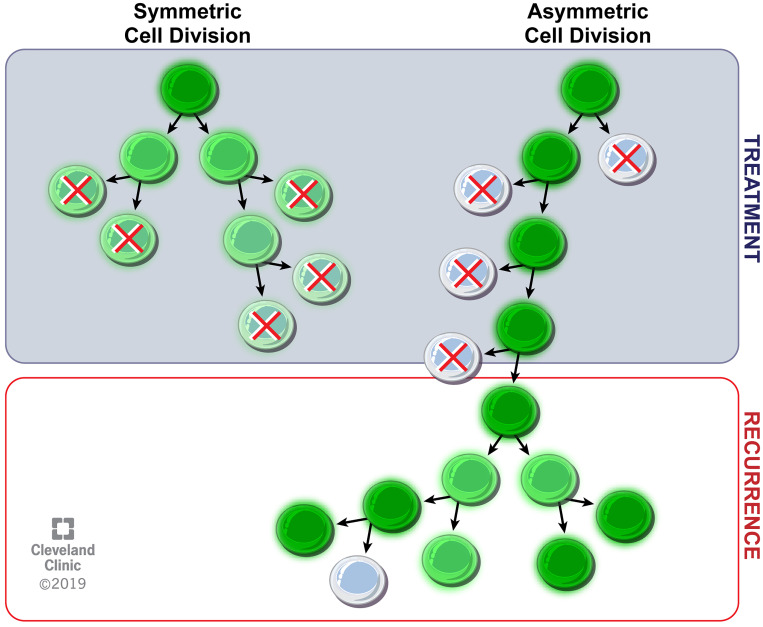

## Introduction

Cancer stem cells (CSCs) drive tumor growth and are resistant to conventional therapies (1, 2). Therapeutic resistance in CSCs has been attributed to multiple mechanisms, including active drug efflux pumps, enhanced DNA repair capacity, slow proliferation rate, and activation of key survival pathways (3–5). While these resistance mechanisms have been identified, the mechanisms by which CSCs emerge, are maintained, and evolve as a result of therapies have yet to be determined. Central to the identity of CSCs is their ability to give rise to all types of malignant cells found in tumors and their ability to maintain a tumorigenic CSC population through self-renewal. CSCs generate such diverse progeny by executing multiple modes of cell division.

Asymmetric cell division (ACD) is a cell division mode to generate cellular heterogeneity while simultaneously maintaining a stem cell population (6). ACD has been observed in multiple advanced cancers (7–10), yet its functional contribution to tumorigenesis is not well understood. Since ACD can enrich fate-determining molecules in one of the emerging daughter cells, we hypothesized that this cellular mechanism may be leveraged in CSCs to generate therapeutically resistant progeny by concentrating prosurvival molecules to one daughter cell at the expense of the other.

We previously demonstrated that CSCs from glioblastoma (GBM), the most common primary malignant brain tumor (11), execute ACD (8). We now show that a functional consequence of ACD is the ability to enrich prosurvival signaling activity by EGFR and nerve growth factor receptor (p75NTR) in 1 daughter cell. In preclinical xenograft models, interfering p75NTR signaling sensitized CSCs to a treatment regimen targeting EGFR, which showed limited clinical efficacy as single agents (12, 13).

## Results

### A lipid-raft reporter informs the mode of cell division and the fate of progeny.

**Figure 1:**
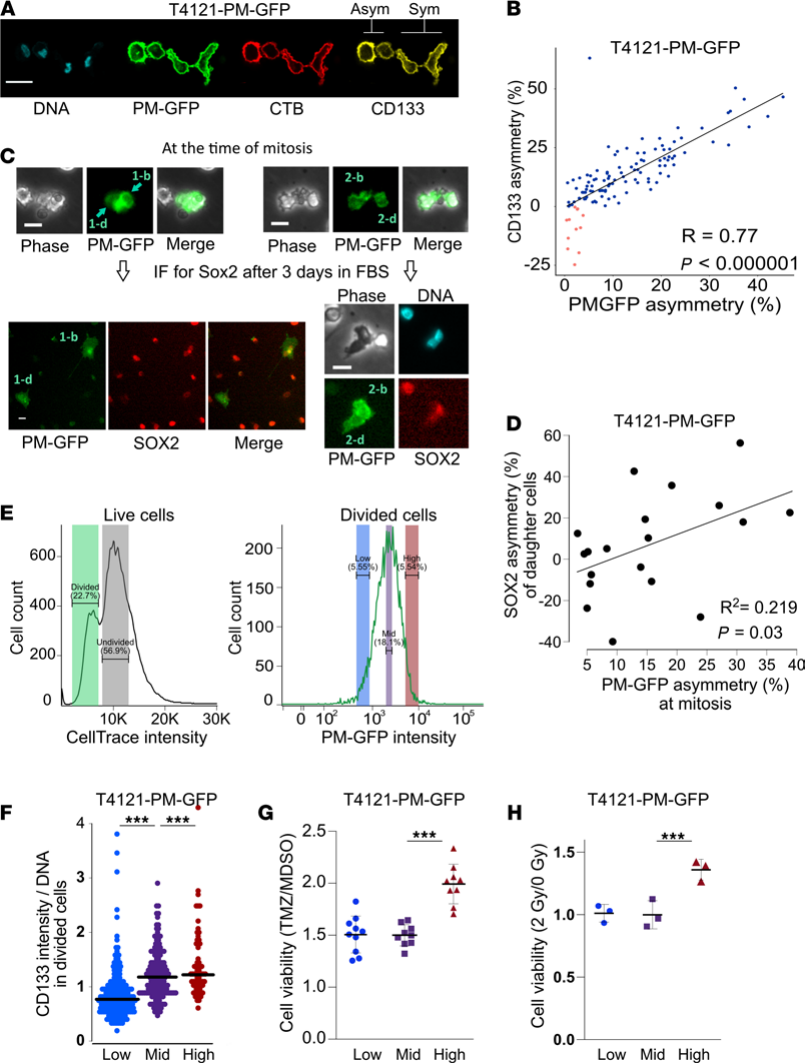
A plasma membrane–green fluorescence protein (PM-GFP) reporter system allows the reliable evaluation of cell division mode and reveals functional differences in asymmetrically divided cells. (**A**) Confocal microscopy captured asymmetrically (left) and symmetrically (right) dividing T4121-PM-GFP cancer stem cells (CSCs) in telophase. Scale bar: 20 μm. CSCs expressing a lipid raft marker, PM-GFP (green), were stained with Hoechst 333342 for DNA (cyan), with cholera toxin B (CTB, red), another marker of lipid rafts, and with a specific antibody for surface CD133 (yellow). (**B**) Quantification of asymmetry percentage during mitosis reveals a significant correlation between asymmetry of PM-GFP and CD133 by Pearson’s correlation coefficient analysis (*P* < 0.00001). Each dot represents 1 cell division. Blue dots indicate divisions with cosegregation of PM-GFP and CD133 to the same progeny. Divisions exhibiting segregation of each marker to opposite progeny are shown in red. (**C**) Time-lapse microscopy recording detected asymmetric PM-GFP inheritance between T4121-PM-GFP CSC daughter cells: darker cell (d) and brighter cell (b), at the time of mitosis. Progeny were traced, and their SOX2 levels were quantified by immunofluorescence after time-lapse recording. Scale bars: 20 μm. (**D**) Pearson’s correlation coefficient analysis demonstrated a significant association between the degree of PM-GFP asymmetry at the time of mitosis and SOX2 expression asymmetry of corresponding progeny at the end of the 3-day -time-lapse microscopy (*P* = 0.03). (**E**) FACS analysis of cells released from S phase synchronization. Once-divided cells (green shaded) exhibited a CellTrace signal intensity that was half the value of the nondivided cells (gray shaded). Gated divided cells were then sorted based on their PM-GFP signal. As asymmetric divisions constituted 10%–15% of divisions in T4121-PM-GFP cells, the top and bottom 5% of PM-GFP cells (PM-GFP–high and PM-GFP–low) were sorted as asymmetrically divided, and the cells in the middle fraction of the PM-GFP distribution (PM-GFP–mid) were selected as progeny of symmetric division. (**F**) CD133 immunofluorescence intensity was quantified for each sorted faction cell normalized by DNA content. PM-GFP–low, PM-GFP–mid, and PM-GFP–high populations expressed CD133 at significantly different levels, with the highest CD133 mean level in PM-GFP–high and the lowest in PM-GFP–low (***P < 0.000001, 1-way ANOVA). Bars indicate mean expression levels. (**G** and **H**) Cell viability of PM-GFP–low, PM-GFP–mid, and PM-GFP–high populations after 3-day exposure to 100 μM temozolomide (TMZ, with 2 biological replicates) (**G**), or 3 days after 2 Gy γ-irradiation (**H**). PM-GFP–high cells had a significantly higher relative viability (mean ± SEM, ***P < 0.000001, 1-way ANOVA).

We previously demonstrated the asymmetric inheritance of CD133, a CSC marker, in a minor fraction of GBM CSC mitoses (approximately 4% during expansion conditions; ref. 8). The frequency of this type of cell division increased under differentiation-inducing conditions (deprivation of growth factors), which also increased the incidence of asymmetric cell fate choice as determined by lineage tracing (8). To establish a direct connection between ACD and differential cell fate determination, we developed a green fluorescence protein–based (GFP-based) reporter for CD133 inheritance at the time of mitosis. Based on the observation that CD133 is enriched in cholesterol-rich lipid rafts (14), we reasoned that a GFP fusion protein containing the N-terminus of Lyn (plasma membrane–green fluorescence protein; PM-GFP) that is enriched in lipid rafts through myristoylation/palmitoylation of its N-terminus (15) would report CD133 distribution between the 2 daughter cells during mitosis. To test the ability of PM-GFP to report modes of cell division in CSCs, we introduced a CMV promoter–driven PM-GFP expression vector into a patient-derived GBM CSC model (T4121) and established a stable cell population of constitutively expressing PM-GFP. We stained PM-GFP–expressing CSCs for lipid rafts with fluorochrome-labeled cholera toxin B (CTB) and for CD133 using immunofluorescence, and we observed that this reporter cosegregated with both lipid rafts and CD133 during ACD (Figure 1A). Quantification of the fluorescent signals of telophase cells demonstrated a correlation between PM-GFP and CD133 asymmetry (Figure 1B), with the majority of cells cosegregating both markers to the same daughter cell. Importantly, the expression of this reporter gene did not adversely impact the self-renewal capacity of CSCs (Supplemental Figure 1A; supplemental material available online with this article; https://doi.org/10.1172/jci.insight.130510DS1). These results suggest that PM-GFP provides reliable reporting of the asymmetric inheritance of lipid rafts and CD133.

As PM-GFP reports the mode of cell division by indicating the degree of asymmetry of lipid raft inheritance during mitosis, we combined this system with time-lapse videomicroscopy–based lineage-tracing analysis to prospectively determine the impact of cell division mode on the cell-fate decision of CSCs. The PM-GFP signal was captured every 30 minutes to determine the degree of asymmetry during mitosis (Figure 1C, top panels, showing a mitotic cell undergoing ACD and a daughter cell on the right side receiving a higher amount of PM-GFP). Phase-contrast images were taken every 3 minutes to trace the migrating daughter cells through the recorded time-lapse images. After the recording, the cells were fixed and stained to assess SOX2 expression as a surrogate for the CSC state (Figure 1C, bottom panel). This approach revealed that the daughter cell that inherited higher PM-GFP at the time of mitosis also eventually expressed elevated SOX2 compared with its counterpart under a differentiation-inducing condition (Figure 1D). This observation indicates that the asymmetry of PM-GFP inheritance, which reflects that of lipid rafts and CD133 cosegregation, prospectively predicts the fate of CSC progeny.

### ACD generates progeny with enhanced therapeutic resistance.

CSCs are resistant to conventional therapies (1, 2). To investigate whether the mode of cell division alters therapeutic resistance of the resulting CSC progeny, we isolated dividing daughter cells generated through symmetric and ACDs using a FACS-based approach. To achieve this, PM-GFP CSCs were synchronized in S phase and labeled with CellTrace dye. The S phase arrested cells with uniform levels of PM-GFP, and CellTrace intensities were enriched by the first round of sorting (Supplemental Figure 1, B and C). The cells were then released from the S phase arrest, and cells that had divided once were captured 15 hours later by the second FACS using a gating for the CellTrace intensity (Figure 1E, left panel). Since the cells arrested in S phase were released into a differentiation-inducing condition that induced ACD incidence up to 10%–15% of the total divisions (8), collecting the once-divided cells with the 5% highest and lowest intensities of PM-GFP likely captured the progeny of ACDs, and the cells with mid PM-GFP levels were likely to be progeny of symmetrically divided CSCs (Figure 1E, right panel). The fidelity of this approach was confirmed by CD133 staining of the sorted populations, revealing that the highest levels of CD133 were detected in PM-GFP–high cells and the lowest CD133 levels in PM-GFP–low cells (Figure 1F). This finding is in accordance with the cosegregation of CD133 and PM-GFP during mitosis (Figure 1, A and B). The progeny with the highest levels of PM-GFP inheritance had increased survival when challenged with GBM standard-of-care therapies temozolomide (TMZ) (Figure 1G) and ionizing radiation (Figure 1H). Taken together, these data demonstrate that ACD generates a population of CSC progeny with an enhanced ability to resist conventional therapies, a CSC phenotype (1, 2), and also provide evidence that CD133-high cells with enhanced therapeutic resistance emerge after an ACD.

### ACD coenriches EGFR and p75NTR.

Lipid rafts are not only enriched in CD133, a CSC marker, but also in many other signaling molecules, including growth factor receptors that are responsible for therapeutic resistance and tumor growth (16). We hypothesized that asymmetric inheritance of lipid rafts marked by PM-GFP results in enrichment of growth factor receptors in the favored daughter cell. We determined the mitotic asymmetry of EGFR and p75NTR and compared it with that of the PM-GFP ACD reporter. We chose these receptors based on their important roles in GBM biology. EGFR is a major driver of malignancy (17) and the focus of a multitude of inhibitory strategies (18). p75NTR facilitates cell infiltration, and its ligand is implicated in GBM progression (19, 20) and is an ongoing interest in targeting its coreceptors (21). Furthermore, high levels of p75NTR expression are associated with poor GBM patient prognosis, with a more pronounced association within the recurrent GBM patients (Supplemental Figure 2).

**Figure 2:**
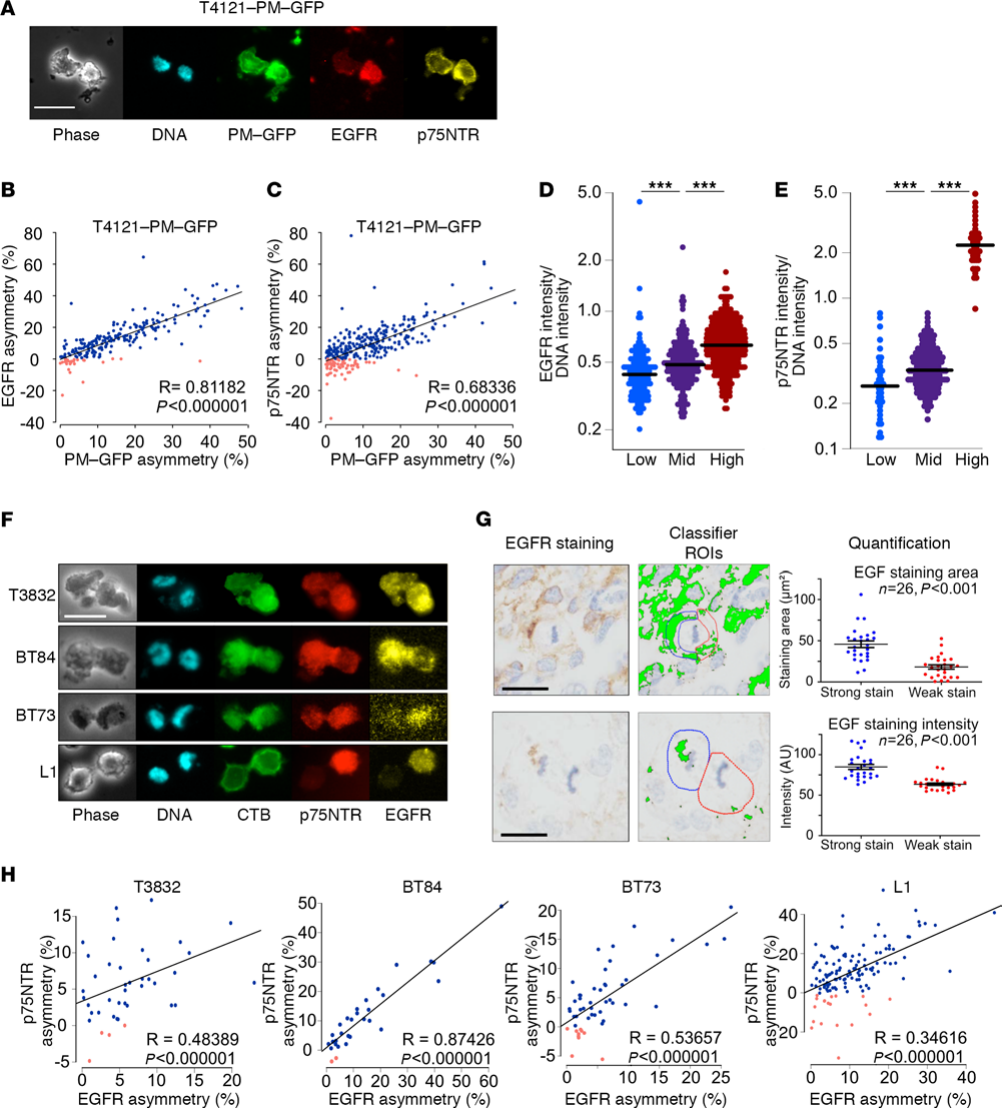
EGFR and p75NTR cosegregate during asymmetric cell division. (**A**) Immunofluorescence staining of an asymmetrically divided T4121-PM-GFP cell in late telophase. Scale bar: 20 μm. PM-GFP (green), EGFR (red), and p75NTR (yellow) are shown, and DNA is stained with Hoechst 333342 (blue). (**B** and **C**) Quantification of percentage of asymmetry during mitosis reveals a correlation between the asymmetry of PM-GFP and EGFR (**B**) and PM-GFP and p75NTR (**C**). Each dot represents 1 cell division. Divisions with cosegregated PM-GFP and EGFR/p75NTR on the same daughter cell are marked in blue. Divisions that exhibited segregation of these markers on opposite daughter cells are marked in red. Calculated Pearson’s correlation coefficient demonstrated a significant association (*P* < 0.000001) between PM-GFP reporter asymmetry and that of EGFR (**B**) and p75NTR (**C**). (**D** and **E**) Immunofluorescence staining quantification of EGFR (**D**) and p75NTR (**E**) expression in sorted symmetrically and asymmetrically divided cells. EGFR/p75NTR signal intensity per cell was normalized to DNA intensity per cell. PM-GFP–low, PM-GFP–mid, and PM-GFP–high populations all expressed significantly different EGFR and p75NTR expression levels, with the highest expression level in PM-GFP–high and the lowest in PM-GFP–low (***P < 0.000001) as calculated by 1-way ANOVA. Bars indicate mean values. (**F**) Immunofluorescence staining of asymmetrically dividing cells in late telophase from 4 different glioma stem cell specimens. Scale bar: 20 μm. CTB, used as lipid raft marker (green); p75NTR (red); and EGFR (yellow) are shown. DNA was stained with Hoechst 333342 (cyan). (**G**) EGFR asymmetry in mitotic cells in human GBM tumors was captured after IHC. Scale bar: 10 μm. After staining signal was classified (green marking), the daughter cell with higher staining was defined by blue region of interest (ROI), and the other with less staining by red ROI. Quantitative analysis on 26 mitotic cells that exhibited asymmetric EGFR staining area (green classifier) and intensity (brown staining under classifier) detected significant difference of EGFR staining between the daughter cells (Mean ± SEM, *P* < 0.001 as calculated by 1-way ANOVA). (**H**) Quantification of asymmetry percentage during late telophase reveals a correlation between asymmetry of EGFR and p75NTR in 4 different non–PM-GFP–expressing glioma stem cell specimens. Divisions where EGFR and p75NTR cosegregated on the same daughter cell are marked in blue. Divisions that exhibited segregation of these markers on opposite daughter cells are marked in red. Pearson’s correlation coefficients were calculated and demonstrated a significant association (*P* < 0.000001) between asymmetry of p75NTR and that of EGFR.

In addition to asymmetric distribution of EGFR, which has been reported in GBM CSCs (22, 23), we found that both EGFR and p75NTR cosegregated with the PM-GFP ACD reporter (Figure 2A). Costaining of EGFR and p75NTR demonstrated that these receptors were most often coenriched in one of the daughter cells during PM-GFP reported ACDs (Figure 2, B and C). To verify the cosegregation of our PM-GFP reporter and these 2 growth factor receptors, we utilized our FACS-based approach to isolate progeny generated through symmetric cell division and ACD. We collected progeny generated through ACD with low or high levels of PM-GFP, as well as those generated through symmetrical division with mid–PM-GFP intensities and immunostained for the growth factor receptors. The expression levels of EGFR (Figure 2D) and p75NTR (Figure 2E) correlated with PM-GFP intensity, indicating that these receptors are cosegregated with PM-GFP during mitosis. To validate these observations in GBM CSC models from multiple GBM patient specimens, we marked lipid rafts with fluorescently labeled CTB and immunostained for EGFR and p75NTR. We detected cosegregation of lipid rafts and these receptors in CSCs from 4 additional specimens (Figure 2F).

To confirm ACD occurrence in vivo, we used IHC and were able to detect mitotic EGFR segregation asymmetry in human GBM specimens (*n*=26, EGFR ACD; Figure 2G). Representative images of human GBM cells in metaphase and anaphase (Figure 2G) indicate higher EGFR immunoreactivity on the left side of the cell. When the signal was digitized (pixels with positive staining were assigned green color to indicate the region of interest for measurement) and quantified, the cell membrane located on the left side was enclosed with a region tool (blue line) that had a higher staining intensity than the right half (Figure 2G). We also verified ACD occurred in vivo using orthotopic xenograft tumors derived from PM-GFP–expressing CSCs. We found asymmetric cosegregation of EGFR with PM-GFP in mitotic cells (Supplemental Figure 2B). These data demonstrate cosegregation of our lipid raft reporter and key growth factor receptors to the same daughter cell during ACD and the presence of ACD in tumors.

### Asymmetric coenrichment of EGFR and p75NTR promotes CSC maintenance.

**Figure 3:**
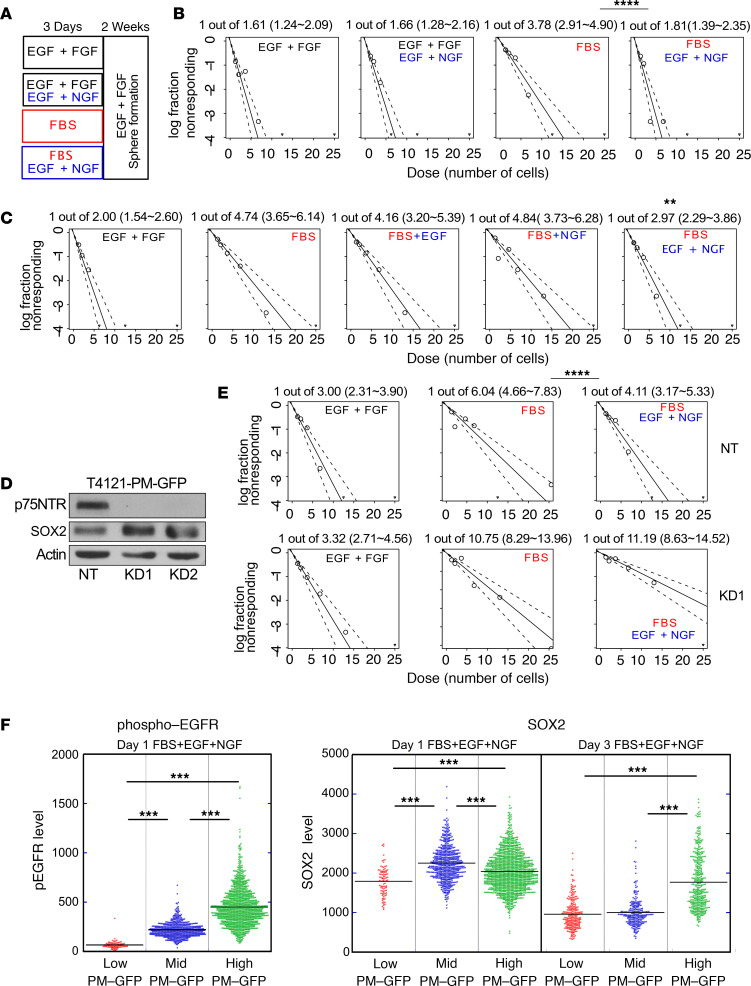
Alteration of the p75NTR axis modifies CSC phenotypes after differentiation. (**A**) Experimental design to assess self-renewing capacity after differentiation induction. T4121-PM-GFP CSCs were subjected to 3 days of pretreatment with either CSC medium (containing epidermal growth factor [EGF] and fibroblast growth factor 2 [FGF2]), CSC medium with the addition of nerve growth factor (NGF), 10% FBS containing differentiation medium without EGF and FGF, or medium with 10% FBS, along with EGF and NGF. The cells were then plated for limiting-dilution assay in CSC medium containing EGF and FGF2 for 2 weeks and assessed for self-renewal (sphere forming) capacity. (**B**) Self-renewal capacity of T4121-PM-GFP cells after 3-day exposure to these conditions. (**C**) Self-renewal capacity of T4121-PM-GFP cells after differentiation using 10% FBS with and without growth factor stimulation (EGF, NGF, or combination). (**D**) Immunoblotting showing expression of p75NTR and SOX2 in T4121-PM-GFP cells expressing p75NTR knockdown shRNAs (KD1 and KD2) compared with nontargeting (NT) shRNA. (**E**) Self-renewal capacity of T4121-PM-GFP cells in stem cell medium (NB) alone, in the presence of 10% FBS (NB + FBS), or with FBS with EGF and NGF together (FBS + EGF + NGF). Nontargeting shRNA (NT) transduced cells were compared with knockdown shRNA (KD1) transduced cells. (**B**, **C**, and **E**) Estimated stem cell frequencies are shown with 95% CI (numbers in parentheses). ****P < 0.000001, **P = 0.0144. (**F**) Expression levels of phospho-EGFR and SOS2 in T4121-PM-GFP cells generated through different modes of cell divisions were determined for individual cells by quantitative immunofluorescence. After cell sorting, divided daughter cells of three groups — cells with asymmetrically depleted PM-GFP (low), symmetrically divided cells with mid levels of PM-GFP (mid), and those with asymmetrically enriched PM-GFP (high) — were cultured in differentiation-inducing serum containing medium supplemented with EGF and NGF. Left panel is quantified phospho-EGFR levels after culturing for a day, and the right panel shows levels of SOX2 expression on day 1 and 3 (**P < 0.001 as calculated by 1-way ANOVA). SOX2 expression levels on Day 3 were analyzed by Wilcoxon test. Each dot represents an individual cell.

Our PM-GFP reports asymmetric coenrichment of EGFR and p75NTR on one of the daughter cells during ACD (Figure 2) and can prospectively predict the phenotypic fate of the cell with elevated SOX2 expression compared with its sister cell (Figure 1C). These observations imply that coenrichment of these growth factor receptors may promote maintenance of the CSC phenotype. To verify this possibility, we assessed the biological importance and interaction of the 2 receptors in CSC maintenance under a serum-based, differentiation-inducing paradigm (24). Exposure to serum for 3 days reduced the number of cells with self-renewal capacity (Figure 3, A and B). While simultaneous stimulation of both EGFR and p75NTR by epidermal growth factor (EGF) and nerve growth factor (NGF) partially prevented the reduction in self-renewing capacity, activation of each receptor alone was not sufficient to override the differentiation-inducing effects of serum (Figure 3, B and C). To confirm the importance of coexpression of the 2 receptors, we knocked down p75NTR (Figure 3D). CSCs tolerated p75TNR knockdown and showed similar self-renewing capacity as those transduced with nontargeting shRNA when sphere-forming capacity was assessed in the medium, supporting CSC maintenance (Figure 3E). However, knockdown of p75NTR attenuated the ability of EGF and p75NTR ligands to override the effect of serum to suppress self-renewal (Figure 3E).

These observations suggest that asymmetrically coinherited receptors during mitosis would benefit one of the daughter cells to maintain self-renewal capacity as compared with the other daughter cell, which would be depleted for these receptors. In order to test this hypothesis, we exposed CSC progeny generated through symmetrical cell division or ACD to differentiation-inducing conditions, together with ligands of both receptors. PM-GFP–expressing CSC progeny inheriting intermediate levels of receptors through symmetrical cell division, and those generated through ACD receiving the lowest or highest levels of receptors, were collected by sequential sorting as previously described (Figure 1 and Supplemental Figure 1). After culturing with a medium containing serum (differentiation inducer), EGF, and NGF (growth factors antagonize action of serum; Figure 3, A and B) for a day, phospho-EGFR (indicative of the activated form of EGFR) signal was highest in the progeny inherited highest levels of receptors through ACD, and it was lowest in the ACD-generated progeny with lowest receptor inheritance (Figure 3F). The activated EGFR level was intermediate in symmetrical cell division–generated progeny inheriting intermediate levels of receptors (Figure 3F). At this time point, however, all 3 groups of CSC progeny maintained similar SOX2 expression (Figure 3F). By 3 days of culture, when serum can substantially suppress CSC self-renewal capacity (Figure 3, A and B), expression of SOX2 was suppressed in the progeny that received intermediate or lowest levels of growth factor receptors, whereas the progeny that inherited the highest levels of receptors through ACD maintained SOX2 expression compared with the other groups (Figure 3F). These data indicate that asymmetric coenrichment of growth factor receptors to one of the daughter cells during mitosis enables a receptor-enriched progeny to better respond to ligands and to promote stem cell maintenance under differentiation inducing conditions.

### p75NTR stimulation restores the signaling activity suppressed by EGFR inhibition.

**Figure 4:**
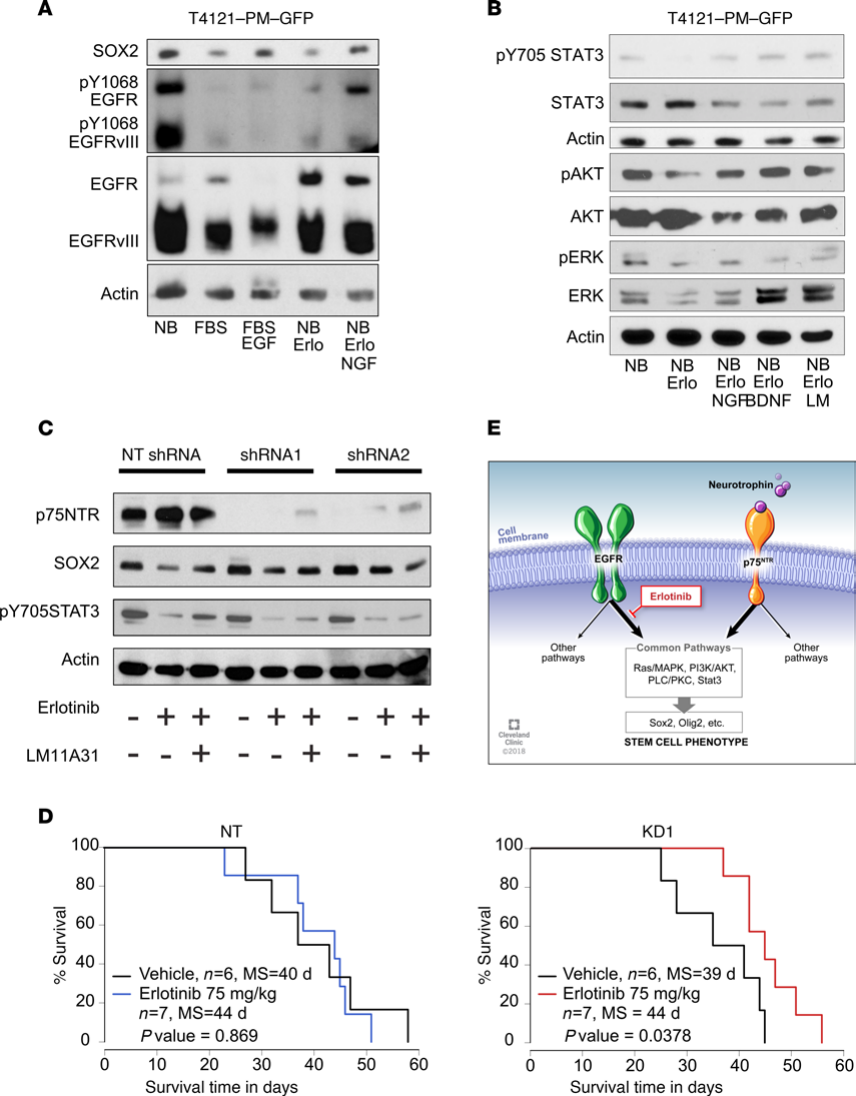
p75NTR signaling modifies response to EGFR inhibition. (**A**) Immunoblotting detected SOX2 expression and EGFR receptor activation in T4121-PM-GFP CSCs after a 3-day treatment with stem cell medium (NB), FBS containing differentiation-inducing medium (FBS), differentiation medium with EGF (FBS + EGF; 20 ng/mL), NB with the EGFR inhibitor erlotinib (Erlo; 3 μM), or a combination of erlotinib and NGF (Erlo + NGF; 100 ng/mL) to stimulate p75NTR. (**B**) Immunoblotting detected STAT3, AKT, and ERK activation in T4121-PM-GFP CSCs after a 3-day treatment with stem cell medium (NB), the EGFR inhibitor erlotinib (NB + Erlo; 3 μM), or a combination of erlotinib and NGF (NB + Erlo + NGF; 100 ng/mL), BDNF (NB + Erlo + BDNF; 100 ng/mL), or the p75NTR ligand LM11A-31 (NB + Erlo + LM; 100 nM) to stimulate the p75NTR. (**C**) Immunoblotting for EGFR receptor activation and SOX2 expression in nontargeting (NT) and knockdown (KD1 and KD2) T4121-PM-GFP CSCs. Cells were treated for 3 days with CSC medium (NB) alone, CSC medium with the addition of the EGFR inhibitor erlotinib (Erlo; 3 μM), or 3 μM erlotinib and the p75NTR ligand, 100 nM LM11A-31 (Erlo+LM). (**D**) Kaplan-Meier plots indicate the survival of the mice that were intracranially implanted with T4121-PM-GFP CSCs that were transduced with nontargeting (NT) shRNA or p75NTR knockdown (KD1) shRNA. The erlotinib treatment group received 75 mg/kg per day (blue and red lines). Animals in the vehicle group were treated with 0.05% methylcellulose solution (black lines). Median survival and *P* value as determined by log rank test comparing the vehicle and erlotinib groups are shown. (**E**) Schematic depiction of the proposed model of signaling that occurs in GBM CSCs upon EGFR and p75NTR cosegregation during ACD. The 2 receptors signal through similar signaling pathways that promote the stem cell phenotype. Upon inhibition of EGFR by erlotinib, ligand-activated p75NTR takes over the downstream stimulation to maintain the stem cell program.

EGFR signaling is activated in the majority of GBM cases, making this receptor a candidate for therapeutic targeting (25, 26). EGFR targeting through inhibition of its kinase activity, however, failed to show therapeutic benefit in clinical trials (17). Based on our current observation of EGFR and p75NTR cosegregation and their reportedly overlapping downstream signaling pathways (27), we hypothesized that signaling activity from p75NTR could compensate for EGFR inhibition. To test this hypothesis, we stimulated cells with NGF, a p75NTR ligand, in the presence of erlotinib, which inhibits EGFR kinase activity. The importance of EGFR signaling was confirmed by the following observations. EGF stimulation restored SOX2 expression that was suppressed by differentiation-inducing FBS treatment. Erlotinib suppressed autophosphorylation of full-length and truncated mutant EGFR, as well as SOX2 expression (Figure 4A). The erlotinib-induced reduction in both autophosphorylation of EGFR Y1086 and SOX2 expression was rescued when cells were stimulated with NGF, which is expressed in the brain (Figure 4A and Supplemental Figure 3A). We assessed known downstream signaling nodes of these receptors and found that the erlotinib-mediated reduction in STAT3 and AKT phosphorylation was overridden by NGF and brain-derived neurotrophic factors (BDNF) (Figure 4B). These neurotrophins stimulate p75NTR and its tropomyosin receptor kinase (Trk) coreceptors, which activate downstream signaling cascades common to those activated by EGFR (28). To examine the role of Trks in restoring the downstream signaling blocked by erlotinib, we used LM11A-31, a chemically synthesized p75NTR ligand that does not activate Trks (29). Similar to the endogenous ligands, this synthetic ligand restored the activating phosphorylation of STAT3 and AKT that was blocked by erlotinib, indicating that the tyrosine kinase activity of Trks is not required. In contrast to STAT3 and AKT, the phosphorylation of another known downstream signaling mediator, ERK, was less affected by the p75NTR ligand (Figure 4B). Importantly, STAT3 is a well-established CSC maintenance signaling node (30) that is activated through EGFR signaling (31). Our results indicate that EGFR inhibition is not sufficient to suppress STAT3 activity when converging p75NTR signaling is activated.

### p75NTR attenuates the therapeutic efficacy of EGFR inhibition.

To further understand the role of the p75NTR receptor in the context of EGFR-targeted therapy, we next treated p75NTR knockdown cells with erlotinib to suppress EGFR kinase activity. While the reduction in p75NTR did not suppress SOX2 expression (Figure 4C) or self-renewal capability of CSCs (Figure 3E), knockdown of this receptor attenuated the ability of p75NTR ligand to rescue SOX2 expression suppressed by erlotinib (Figure 4C). To determine whether p75NTR knockdown increased the sensitivity of CSCs to erlotinib, we assessed the efficacy of erlotinib in a preclinical orthotopic xenograft model. We first determined the minimal effective dose of erlotinib in vivo by evaluating a dose range from 5 to 100 mg/kg, similar to a previously reported range (32). We found that 100 mg/kg significantly increased the survival of tumor-bearing mice, while lower doses did not increase survival (Supplemental Figure 3B). We reasoned that, if p75NTR compensates for EGFR function that is suppressed by erlotinib, as we observed in vitro (Figure 4, A–C), then xenograft tumors originating from p75NTR-knockdown CSCs would become susceptible to erlotinib at a dose that did not affect the tumorigenicity of control CSCs. Indeed, a suboptimal dose of erlotinib, 75 mg/kg, increased the survival of mice bearing p75NTR-knockdown tumors but not the survival of mice with control tumors (Figure 4D and Supplemental Figure 3C). These results demonstrate that the p75NTR signaling axis compensates for EGFR signaling to override the therapeutic efficacy of EGFR inhibition (Figure 4E). These signaling networks are enriched in one of the CSC progeny as a result of ACD, suggesting that ACD has the capacity to generate therapeutically resistant progeny at the expense of their sister cell.

## Discussion

ACD is an essential cell division mode that enables simultaneous maintenance of a stem cell population and the generation of differentiated progeny during embryogenesis, organogenesis, tissue homeostasis, and tissue regeneration (6, 33, 34). ACD has been reported in many advanced cancers, but the importance of ACD during tumorigenesis has yet to be fully elucidated. Technical challenges such as difficulty tracking the fate of daughter cells after different modes of cell division and/or the lack a quantitative approach to determine cell fate choice have prevented studies from elucidating the contribution of ACD to tumorigenic processes. While cell fate tracking has demonstrated dynamic evolution in cancer and therapeutic response, this has not been fully linked to cell fate choice. Moreover, in many studies, ACD is often defined retrospectively based on 2 different phenotypes of the progeny, and this type of retrospective view hampers prospective mechanistic analysis (8, 35). The current work represents a new opportunity to investigate ACD in a prospective manner by virtue of a fluorescent protein–based reporter of asymmetric mitotic inheritance of key signaling molecules. This reporter system enables the previously unfeasible investigation of the impact of ACD on cell-fate decisions, as well as the FACS-based collection of the large cell numbers required for molecular and phenotypic characterization of ACD progeny.

Previous work from our group and others has shown that ACD is not the dominant mode of cell division used by CSCs during tumor growth (8, 36). These findings have also been confirmed using mathematical modeling to suggest an evolutionary disadvantage for ACD (23, 37–42). However, these assessments have not been done in the context of the selective pressures induced by therapies or during biological processes involved in tumor progression. Our current findings demonstrate that ACD becomes advantageous during therapeutic stress by generating a daughter cell with enhanced capacity to withstand therapies. This ACD would not contribute to overall tumor growth but would rather help to preserve a population of cells with a survival advantage that subsequently drives tumor recurrence. While we focused on CSC maintenance in this context, expansion to other key phenotypes — such as invasion, which in another reported function of p75NTR (19) — would be an important direction for future studies.

ACD generates progeny with different phenotypes through asymmetric inheritance of cell fate–determining molecules at the time of mitosis. The current study demonstrates experimentally that EGFR and p75NTR were asymmetrically coenriched to one of the progeny of a CSC undergoing ACD and 2 receptors synergized to protect the self-renewing capacity of a favored CSC daughter cell from differentiation pressure. Interestingly, the coexpression of these receptors is not clearly apparent when assessing mRNA expression of bulk tumor samples in publically available databases, such as the cancer genome atlas (TCGA; https://www.genome.gov/Funded-Programs-Projects/Cancer-Genome-Atlas) and Chinese glioma genome atlas (CGGA; http://cgga.org.cn/), using a GlioVis tool, Corr-Two (http://gliovis.bioinfo.cnio.es/, data not shown). However, our data reveal that, at the single-cell level, coenrichment of these receptors during ACD would benefit the maintenance of self-renewing capacity of one of the progeny.

Coenrichment of these receptors is also expected to benefit CSC maintenance during EGFR-targeted therapy, as p75NTR stimulation restores downstream signaling activities that are suppressed by EGFR inhibition. This paradigm may be useful when designing and assessing the efficacy of pathway-specific inhibitors, such as those targeting EGFR activity, which may require concomitant neutralization of other signaling pathways to achieve a durable therapeutic response. The importance of such an approach is exemplified by the coinheritance of other types of growth factor receptors, such as Met and PDGFRα, with our PM-GFP system (Supplemental Figure 4A). Indeed, coactivation of other receptor tyrosine kinases has been implicated as a mechanism of resistance to EGFR-targeted therapy in GBM (43). These findings highlight the priority for future studies, including the assessment of additional growth factor receptors in the context of ACD and the development of therapeutic resistance, as this may inform additional targeting strategies.

Our finding that a synthetic p75NTR ligand without Trk stimulation activity can restore EGFR signaling reveals a potentially novel, p75NTR-dependent, receptor tyrosine kinase–independent mechanism of resistance against EGFR-targeted therapy. Interestingly, there was a difference in the extent of SOX2 rescue between p75NTR ligands NGF and BDNF with EGFR inhibition, and this highlights the need for a more in-death assessment of not only p75NTR-NGF/BNDF, but also other receptor-ligand interactions. While these findings provide a potentially new function for p75NTR in CSCs, p75NTR reduction itself had limited impact on ACD (Supplemental Figure 4B), which likely is a reflection of the complexity of p75NTR in CSCs and warrants further investigation.

Cellular heterogeneity within tumors has been implicated in tumor therapeutic resistance (43–45). The net fitness of the tumor cannot be determined solely by that of tumorigenic cells such as CSCs or the most proliferative cells in the tumor; a dynamic interaction among the different types of tumor cells and their interaction with stromal cells are also critical factors. Furthermore, reciprocal crosstalk between CSCs and more differentiated tumor cells may contribute to tumor growth (20, 46). In this context, ACD may contribute to overall tumor growth by generating heterogeneous populations of cells that form a mutually beneficial paracrine network involving BDNF, a p75NTR ligand. Analysis of cell division mode should also be expanded to non–stem cancer cells that can revert to a CSC phenotype as a result of chemotherapy or microenvironment-induced stresses.

While these studies provide insight into the role of ACD in therapeutic resistance and CSC fate choice, the underlying fundamental molecular mechanisms of ACD have yet to be determined, and this represents a limitation of this work. Long-noncoding RNAs, as well as transcription factors (10, 47), are asymmetrically distributed during CSC mitosis, and these molecules may regulate the mode of cell division. Another consideration for ACD could be asymmetry of cell volume or morphology that could impact a variety of cellular processes and organelle distribution (48–50). In our GBM CSCs, we assessed ACD by CD133 expression compared with cytoplasmic GFP expression as a read out of cell size. While there was a correlation between GFP expression and CD133, there were examples of ACD where cell size was identical (data not shown). These findings indicate that there may be additional subsets of ACD based on cell size and a need for interrogating such additional cellular heterogeneity. Limited reports addressed the molecular mechanisms driving ACD (51), and this is a priority area for future studies. Another limitation of this current work is the direct assessment of signaling changes in vivo and could be the focus of future studies that could potentially leverage single-cell approaches (including mass cytometry TOF and single-cell RNA sequencing). Asymmetrically generated progeny showed differential sensitivity to TMZ and radiation, standard-of-care therapeutics for GBM. Further studies are required to establish the molecular mechanism downstream of ACD that provides therapeutic resistance. Such studies will reveal exploitable targets to enhance the efficacy of conventional treatments. The cell division reporter and sorting strategies we describe here provide a critical platform to perform such studies.

## Methods

Detailed information on reagent and resources utilized in this study is available in Supplemental Table 1.

### Xenograft maintenance

Established GBM xenografts (T4121, T3832, T4302, BT84, BT73, and L1) were previously reported (3, 22, 52) and were obtained via a material transfer agreement from Duke University, University of Florida, and the University of Calgary, where they were originally established under IRB-approved protocols that facilitated the generation of xenografts in a deidentified manner from excess tissue taken from consented patients. For experimental studies, GBM cells were dissociated from established xenografts under Cleveland Clinic–approved IACUC protocols. Xenografts were passaged in immunodeficient NOD.*Cg-Prkdc^scid^Il2rg^tm1Wjl^*/SzJ (NSG) mice (obtained from The Jackson Laboratory) to maintain tumor heterogeneity. Six-week-old female mice were unilaterally injected s.c. in the flank with freshly dissociated human GBM cells, and animals were sacrificed by CO_2_ asphyxiation and secondary cervical dislocation when tumor volume exceeded 5% of the animal’s body weight.

### CSC isolation

Xenografted tumors were dissected and mechanically dissociated using papain dissociation kits (Worthington Biochemical Corporation), and cells were cultured overnight in Neurobasal medium (Invitrogen) supplemented with B27 (Invitrogen), 1% penicillin/streptomycin (Invitrogen), 1 mM sodium pyruvate, 2 mM L-glutamine, 20 ng/mL EGF (R&D Systems), and 20 ng/mL FGF-2 (R&D Systems) in a humidified incubator with 5% CO_2_. CSCs were enriched using the CD133 Magnetic Bead Kit for Hematopoietic Cells (CD133/2; Miltenyi Biotec) and cultured in supplemented Neurobasal medium. This enrichment method reliably enriches CSCs that have increased self-renewal compared with their non-CSC counterparts (3, 52). Cells were cultured in supplemented Neurobasal medium as sphere cultures or as adherent cultures on plates coated in Geltrex (Invitrogen; a laminin-rich extracellular matrix) until the day they were used. To induce differentiation, adherent cultures on Geltrex-coated plates or coverslips were exposed to 10% FBS (Gibco) containing Neurobasal medium supplemented with B27 but without growth factors.

### Intracranial cell injection and erlotinib treatment

Five- to 8-week-old male or female NSG mice were anesthetized using isoflurane and positioned for intracranial injection using a stereotaxic frame (Kopf Instruments). A total of 5 μL of a single-cell suspension of GBM CSCs was injected into the left striatum at a concentration of 10,000 cells/animal. Two weeks after injection, animals were randomized into treatment and control groups. Daily gavage with 100 μL of either 0.5% methylcellulose (Sigma-Aldrich, vehicle group) or a suspension of erlotinib (Cayman Chemical) in 0.5% methylcellulose (erlotinib group) was performed for 4 weeks. Animals were monitored and euthanized when neurological symptoms developed. For the experiments in Supplemental Figure 3, B and C, female mice were used. For the experiments in Figure 4D, male mice were used.

### PM-GFP expression in CSCs

A PM-GFP plasmid from Addgene (plasmid 21213) was linearized by digestion with the restriction enzyme NruI and transfected into T4121 CSCs using lipofectamine. A stable resistant population was selected with G418 (MP Biomedicals, 1 mg/mL) and sorted using FACS to enrich for GFP^+^ cells. A PM-GFP–expressing stable population was maintained in medium containing a reduced amount of G418 (0.3 mg/mL).

### Mitotic shake-off

To analyze protein expression on daughter cells at the time of mitosis, we enriched for mitotic cells using mitotic shake-off. GBM CSCs were cultured adherently as a monolayer on Geltrex-coated plates. The cells were synchronized in S phase using the addition of 2 mM thymidine to the medium for 12 hours. After synchronization, the cells were released into thymidine-free medium for 12–15 hours to allow the cells to progress through the cell cycle and reach mitosis. At this point, the plates were subjected to gentle vortexing to allow the rounded-up mitotic cells to detach from the plates. The detached cells were washed from the plate and centrifuged at 200 xg for 5 minutes at room temperature onto poly-lysine–coated coverslips. The cells were then promptly fixed with 4% paraformaldehyde and stained.

### Time-lapse fate-decision tracing

GBM CSCs (T4121) and CSCs expressing PM-GFP (T4121-PM-GFP) were plated adherently onto Geltrex-coated 6-well plates as a mixed culture at a ratio of 1:5, with a total of 200,000 cells/well. The cells were synchronized in S phase using the addition of 2 mM thymidine to the media for 12 hours. The cells were then released into CSC medium with 10% FBS but without EGF/FGF to increase the rate of ACD (based on previously published data, ref. 8). Using a sterile needle, a straight 0.5 cm scratch was made at the bottom center of each well. Time-lapse microscopy was then initiated using a Leica CTR6500 microscope with Tempcontrol Digital set-up at 37°C in humidified air with 5% CO_2_, with phase images taken every 5 minutes and green fluorescence captured every 30 minutes to avoid phototoxicity and photobleaching. Acquisition of images was performed using Leica LAS X Life Science software. For each well, 6 fields of view adjacent to the scratch were captured. After 72 hours, the time-lapse was stopped, and the cells were promptly fixed using 4% paraformaldehyde in PBS and subjected to immunofluorescence staining for SOX2. The staining was then reviewed using a Leica DM5000B microscope equipped with a Leica DFC310 FX Digital Color Camera. Images from time-lapse microscopy were exported as .tiff format and analyzed using NIH ImageJ. The exact locations and the individual cells captured by time-lapse imaging were identified using the scratches on the bottom of the wells as reference landmarks. Digital images of the staining were quantified to determine the levels of SOX2 expression in each progeny and correlated with the brightness of PM-GFP that each daughter cell received during mitosis.

### FACS-based isolation of divided cells

To enrich for mitotic cells, we synchronized GBM CSCs (approximately 100 million cells) cultured as spheres at G1/S phase border by adding 2 mM thymidine-containing CSC medium for 12 hours. The spheres were then dissociated into single cells using Accutase (Invitrogen), counted, and labeled with CellTrace Far Red dye (Invitrogen) by suspending the cells in 40 mL of media with 25 μL of CellTrace dye per 100 million cells for 20 minutes at 37°C. The cells were then pelleted and released into CSC medium without thymidine for 6 hours to let them progress to mid–S phase of the cell cycle. The cells were then suspended in FACS buffer (CO_2_ – independent medium [Invitrogen] + B27) containing 2 mM thymidine to prevent further progress through the S phase and subjected to FACS at 4°C over the next 6 hours to isolate a homogeneous population in terms of green and Far Red intensity. Subsequently, the sorted cells were simultaneously released into Neurobasal medium containing 10% FBS and B27 without thymidine to increase the rate of ACD (based on previously published data, ref. 8). After 15 hours, a significant percentage of cells had undergone 1 mitosis, at which point the cells were subjected to a second FACS: live cells were gated based on CellTrace Far Red intensity to collect only the cells that had divided once (Figure 1D), and they were gated for the bottom and top 5% to collect asymmetrically divided cells and for a narrow range in average PM-GFP intensity to select for symmetrically divided daughter cells. Isolated cell populations were further subjected to immunofluorescence staining and functional proliferation assays. For immunofluorescence staining, the cells were centrifuged at 200xg for 5 minutes at room temperature onto Geltrex-coated cover slips and incubated for 1–2 hours to allow the cells to attach prior to fixing.

### Immunofluorescence and immunohistochemical staining

Cells for immunofluorescence staining were fixed on cover slips in 6-well plates using 4% paraformaldehyde in PBS for 15 minutes, followed by rinsing with PBS. Fixed cells were blocked with 2% donkey serum (MilliporeSigma) for 1 hour. For EGFR and p75NTR staining, we permeabilized cells with 0.01% Triton added to the blocking solution. The samples were then incubated overnight at 4°C with primary antibodies against CD133, EGFR, and/or p75NTR; washed 3 times with PBS; and incubated for 1 hour at room temperature with secondary antibodies: DyLight-649–conjugated donkey anti–rabbit IgG, Cy3-conjugated donkey anti–mouse IgG, and DyLight-649–conjugated donkey anti–mouse IgG (Jackson ImmunoResearch). The samples were washed 3 times with PBS and incubated for 15 minutes in PBS containing Hoechst 33342 (100 ng/mL) and/or Alexa-488– or Alexa-594–conjugated CTB. The cover slips were then mounted on slides in gelvatol mounting medium (PVA [MilliporeSigma], glycerol [MilliporeSigma], sodium azide [Thermo Fisher Scientific], and Tris-Cl [pH 8.5]) and subjected to fluorescence microscopy using a Leica DM5000B microscope equipped with a Leica DFC310 FX Digital Color Camera. Images were captured at 40× magnification using a dry objective. For IHC, formaldehyde-fixed paraffin embedded tissue specimens from 19 GBM patients were screened for their fraction of mitotic cells, and the tumor with the highest fraction was selected for further investigation. Tissue sections of 3 μm were cut on a microtome and were subject to deparaffinization, blocking of endogenous peroxidase, and heat-induced epitope retrieval with Protease 1 (Ventana Medical systems) for EGFR and TRIS low-pH buffer for p75NTR. Tissue sections were then stained with primary EGFR antibody, followed by detection with the OptiView-DAB detection system on the Ventana Discovery Ultra staining platform, or they were stained with primary p75NTR antibody using the EnVision FLEX DAB detection system on the Dako Omnis staining platform. Images of mitotic cells were acquired using a Leica DM6000B microscope with an Olympus DP72 camera at 100× magnification. The digital images were imported into the Visiopharm software (Visiopharm), and a threshold-based classifier was created to identify positive immunostaining (green label) in the images. Regions of interest (ROI) were manually outlined in each image, and staining intensity and area in each ROI was quantified by the software. A total of 26 EGFR ACDs was identified and quantified.

### Cell proliferation analysis to determine the effect of therapeutics

To assess the effect of TMZ on proliferation, symmetrically and asymmetrically divided cells derived from GBM CSCs were isolated using the FACS-based approach and plated into 96-well plates at a concentration of 2000 cells/well in 100 μL of either CSC medium with 100 μM TMZ (Santa Cruz) or DMSO (1:1000) in CSC medium as a control, with 8 wells per condition. After 3 days of incubation at 37°C with 5% CO_2_, proliferation was assessed using Cell Titer Glo; 100 μL of reagent was added per well and incubated in the dark for 15 minutes. Luminescence was registered using a Victor 3 multi-well plate reader (PerkinElmer). The experiment was repeated twice.

To analyze the effect of radiation, CSC progeny generated via different modes of cell division were isolated using the FACS-based approach and plated into 96-well plates at a concentration of 2000 cells/well in 100 μL of CSC medium. The cells were then irradiated with a total dose of 0 or 2 Gy using a Shepherd Cs137 irradiator, with 6 wells per condition. After 3 days of incubation at 37°C with 5% CO_2_, proliferation was assessed using Cell Titer Glo; 100 μL of reagent was added per well and incubated in the dark for 15 minutes. Luminescence was registered using a Victor 3 multiwell plate reader.

The effect of erlotinib was determined as follows: GBM CSCs were plated in 96-well plates at a concentration of 2000 cells/well in 100 μL of either CSC medium with 0.3–80 μM erlotinib or DMSO (1:1000) in CSC medium as a control, with 3 wells per condition. After 3 days of incubation at 37°C with 5% CO_2_, proliferation was assessed using Cell Titer Glo; 100 μL of reagent was added per well and incubated in the dark for 15 minutes. Luminescence was registered using a Victor 3 multi-well plate reader.

### Self-renewal assay

To determine the self-renewal capacity of GBM CSCs, 500,000 cells were plated adherently as a monolayer using Geltrex-coated 6 cm tissue culture plates. Cells were cultured under the following conditions for 3 days: NB, CSC medium; NB + EGF + NGF, CSC medium with the addition of extra 20 ng/mL EGF and 100 ng/mL NGF; FBS, Neurobasal medium with 10% FBS, L-glutamine, B27, penicillin/streptomycin, and sodium pyruvate; FBS + NGF, FBS medium with 100 nM NGF; LM, CSC medium with 100 nM LM11A-31 (a synthetic p75NTR ligand; Sigma-Aldrich); FBS + LM, FBS medium with 100 ng/ml LM11A-31; Erlo, CSC medium with the addition of 3 μM erlotinib; Erlo + NGF/BDNF/LM, CSC medium + 3 μM erlotinib + 100 ng/ml NGF/BDNF + 100nM LM. After 3 days, cells were dissociated and plated in 96-well suspension plates at 100, 50, 25, 12, 6, and 3 cells per well for limited-dilution analysis in 100 μL of CSC medium. Two plates per condition were used. After 14 days, wells containing spheres were counted, and the frequency of the cell with self-renewal capacity was analyzed using an online tool (http://bioinf.wehi.edu.au/software/elda/) (53).

### Immunoblotting

GBM CSCs were collected from adherent monolayer cultures, and whole cell lysates were made in a lysis buffer containing 10% NP-40 (MilliporeSigma), 1 mM EDTA, 150 mM NaCl, 10 mM Tris-Cl [pH 7.5], supplemented with protease and phosphatase inhibitor cocktails (MilliporeSigma). Protein expression was analyzed by immunoblotting for expression of phospho-EGFR (Y1068, 1:1000, Cell Signaling Technology), total EGFR (1:1000, E235, Abcam), SOX2 (1:500, R&D Systems), p75NTR (1:1000, Cell Signaling Technology), phospho-STAT3 (1:1000, Y705, Cell Signaling Technology), STAT3 (1:1000, Cell Signaling Technology), pAKT (1:1000, Cell Signaling Technology), AKT (1:1000, Cell Signaling Technology), pMAPK (1:500, Cell Signaling Technology), and MAPK (1:1000, Cell Signaling Technology). Anti–β-actin (1:5000, Santa Cruz Biotechnology Inc.) was used as a loading control. See complete unedited blots in the supplemental material.

### Lentivirus preparation and p75NTR knockdown

Using Biotool DNA transfection reagent (bimake.com), 293T cells were cotransfected with ps.PAX2, p.MD2.G (Addgene), and lentiviral vectors: nontargeting control (PLK0.1) or p75NTR-targeting MISSION shRNA constructs (MilliporeSigma, TRCN0000058153 and TRCN0000058153). The medium was changed 8 hours after transfection, and viral supernatants were collected 12, 24, and 36 hours later. Viral particles were concentrated using polyethylene glycol precipitation and stored at –80°C. T4121-PM-GFP CSCs were infected with the concentrated viral supernatants, selected with 2 mM puromycin for 48 hours. After knockdown was confirmed by immunoblotting, cells were used for further experiments.

### Statistics

#### Asymmetry quantification.

To assess asymmetry in the expression of markers between daughter cells, we developed an ImageJ (NIH) macro for batch digital image processing of CSCs in late telophase, as described previously (8). The macro automatically measures the fluorescence intensity of manually outlined daughter cells and background fluorescence. To quantify the percent asymmetry between daughter cells, a web-app called Asymmetry was built using R language and the packages shiny, ggplot2, cowplot, dplyr, and ggExtra. The app quantified the percent asymmetry of each marker using the following formula:

### ([Cell1Intensity – Background1] – [Cell2Intensity – Background2]) × 100/([Cell1Intensity – Background1] + [Cell2Intensity-Background2]) = %Asymmetry

Pearson’s correlation was calculated to determine the significance of cosegregation between molecules.

#### Immunofluorescence intensity quantification.

Using high-throughput automated single-cell imaging analysis (HASCIA), which was described previously (54), the expression of growth factor receptors on FACS-sorted daughter cells was analyzed. First, the HASCIA image processing script and ImageJ v1.52k were used to obtain single-cell measurements of marker intensity. Then, using the HASCIA web-app, the expression was normalized to DNA intensity, and relative expression difference between groups was assessed using a 2-tailed *t* test.

#### Statistical tests.

*P* < 0.05 was considered significant, and as specified in the text, 2-tailed Student’s *t* test, 1-way ANOVA, Wilcoxon, Pearson’s correlation, or log rank tests were performed to calculate statistical significance. Individual *P* values are detailed in the text and figure legends. The correlation analyses for asymmetry were done using Pearson’s correlation test, and statistical significance for the limiting dilution analyses for sphere formation was calculated using the ELDA online tool described above (χ^2^ analysis). Data analysis was done using Office Excel 2013 (Microsoft), KaleidaGraph Version 4.1 (Synergy Software), and custom R scripts in RStudio using packages survival and limdil, as well as HASCIA.

#### Study approval.

As described above, mouse xenograft experiments were conducted according to the approval from the IACUC at the Cleveland Clinic. Human GBM cells (T4121, T3832, T4302, BT84, BT73, and L1) were obtained from Duke University, University of Florida, and the University of Calgary through material transfer agreements. These xenograft models were originally established under IRB protocols of each institute. For histological assessments, GBM specimens were collected retrospectively from deceased patients’ paraffin embedded blocks according to the protocol approved by Regional Scientific Ethical Committee (Regionshuset, Denmark). The Danish Data Inspection Authority (approval number 16/11065) and the Regional Scientific Ethical Committee of the Region of Southern Denmark (approval number S-20150148) approved use of the human tissue specimens.

## Author contributions

MH, APC, BWK, and JDL designed the study. MH, APC, DJS, AMK, SM, and NA performed experiments and collected the data. MH, APC, AMK, WDP, and SM analyzed the data. MH, APC, and JDL wrote the manuscript. MH, APC, DJS, AMK, BWK, and JDL reviewed and edited the manuscript.

## Supplementary Material

Supplemental data

## Version 1 12/22/2020

In-Press Preview

## Version 2 02/08/2021

Electronic publication
