# Supplementary material for: Asymmetric cell division promotes therapeutic resistance in glioblastoma stem cells
Source: JCI Insight. 2021 Feb 8;6(3):e130510. doi: 10.1172/jci.insight.130510 (PMC7934841; doi:10.1172/jci.insight.130510)
Supplement: Supplemental data [file jciinsight-6-130510-s063.pdf]

Supplemental figure 1

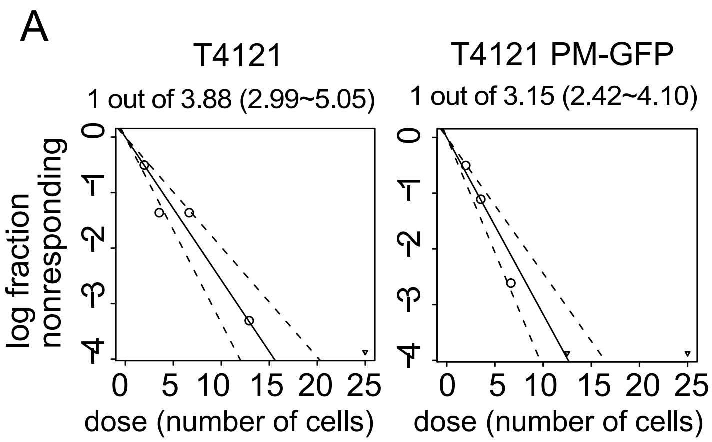

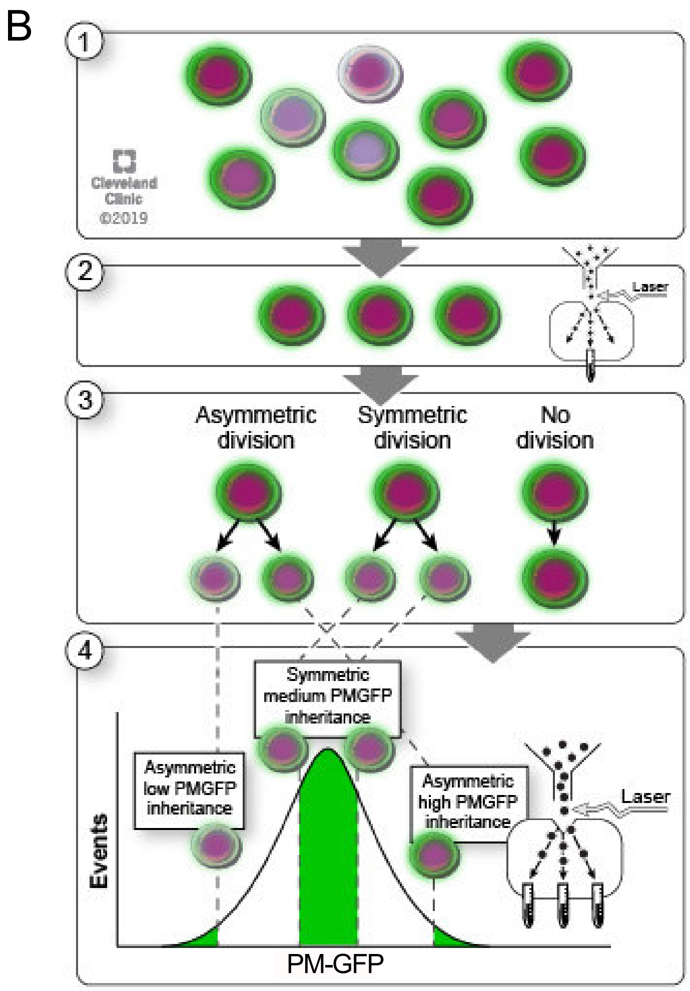

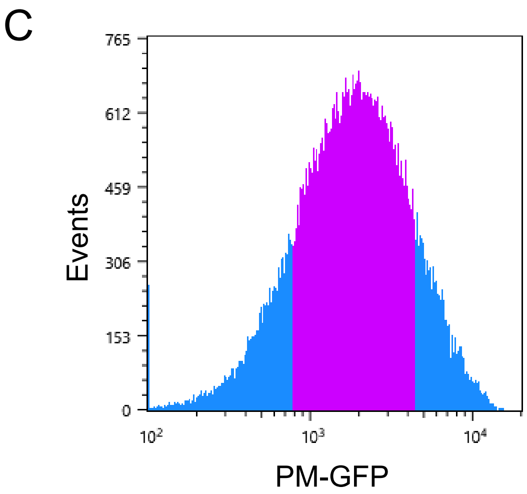

Supplemental figure 2

A

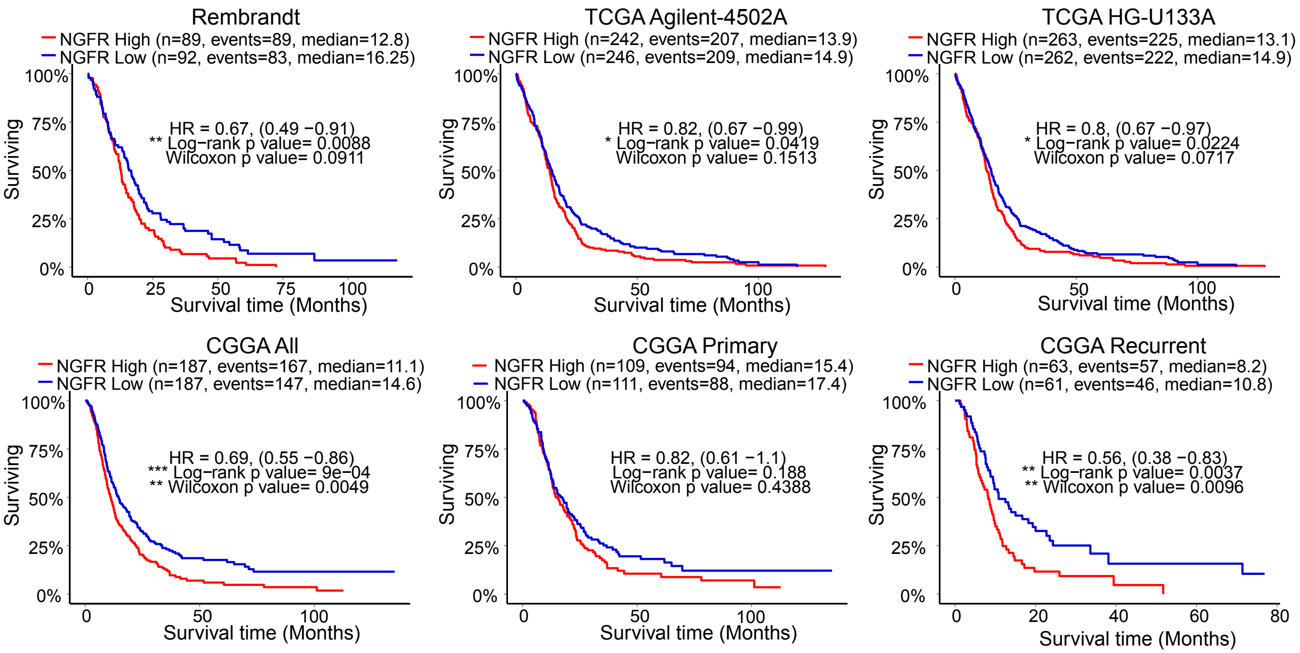

B

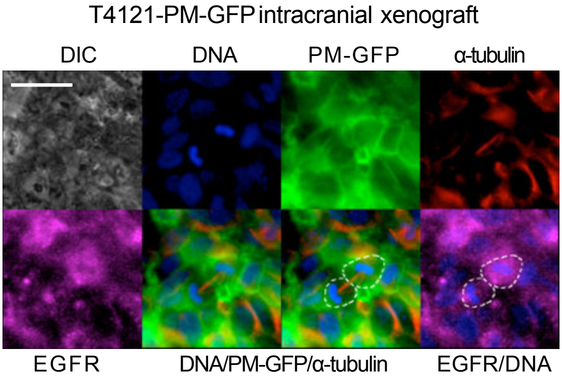

Supplemental figure 3

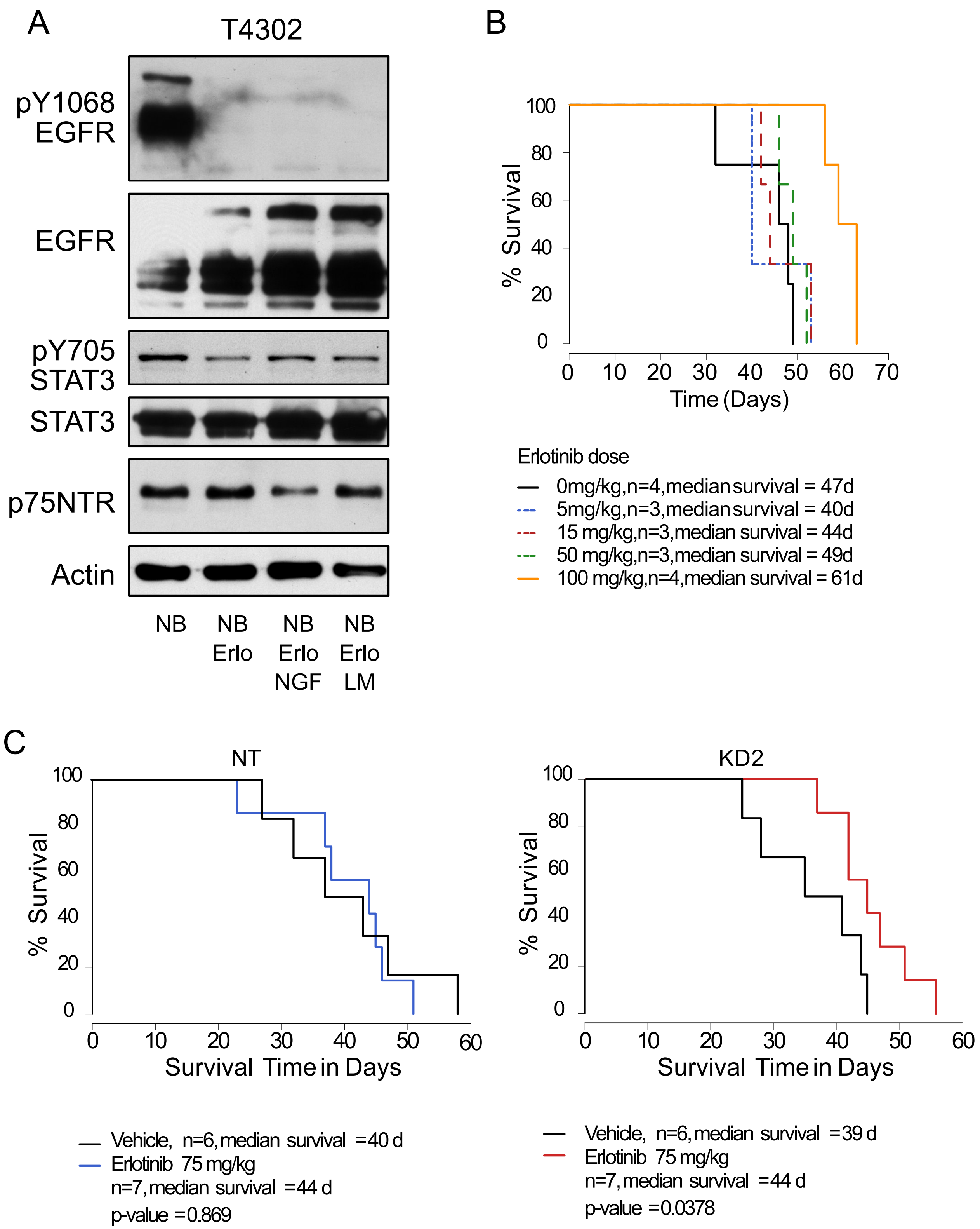

Supplemental figure 4

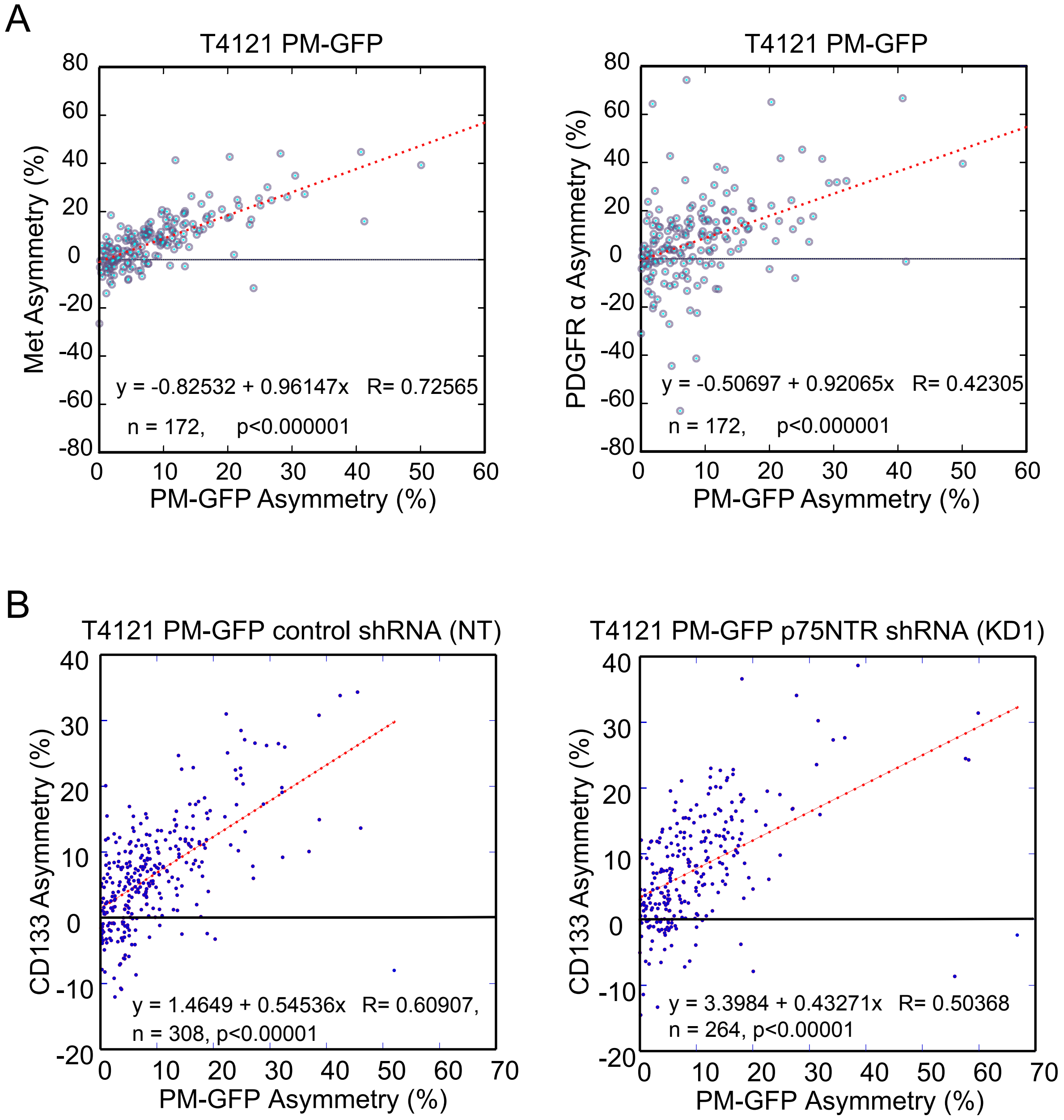

Figure 3, D

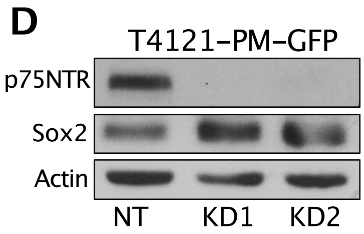

p75NTR

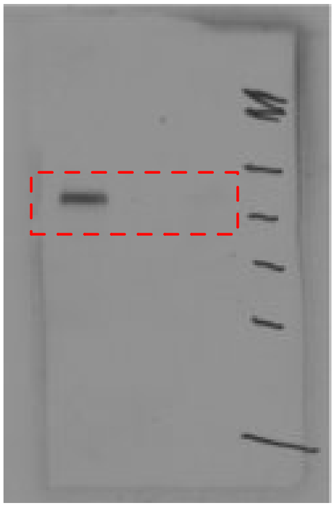

Sox2

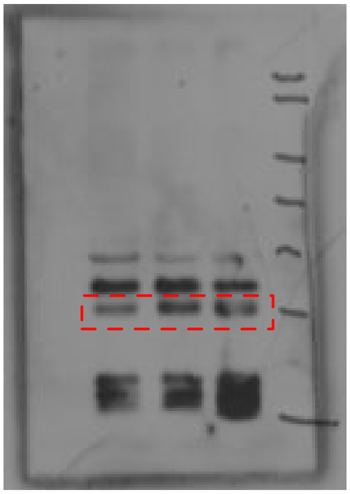

Actin

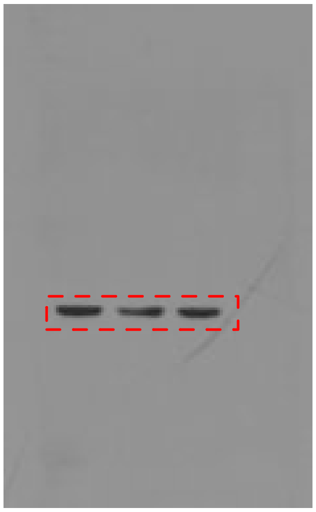

Figure 4A

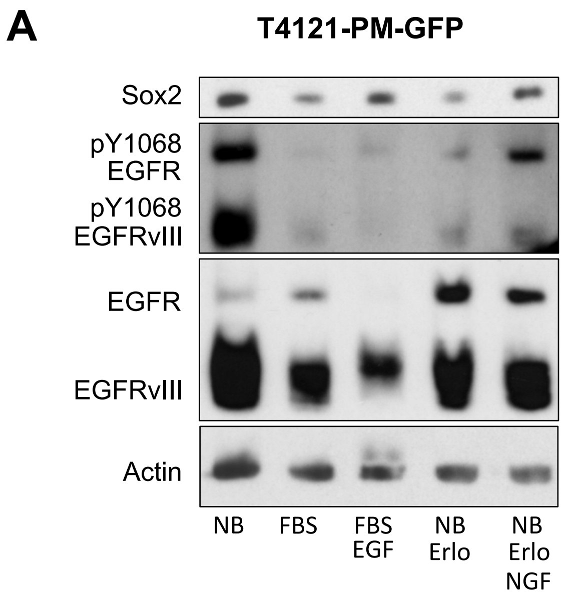

Sox2

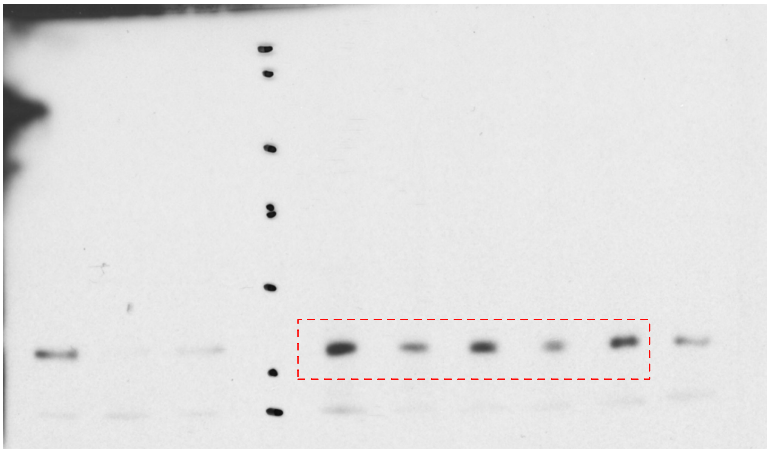

pY1068-EGFR/EGFRvIII

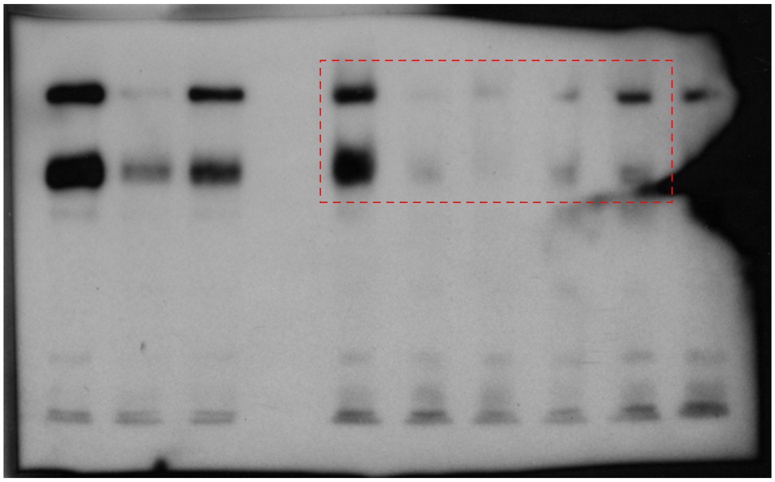

EGFR/EGFRvIII

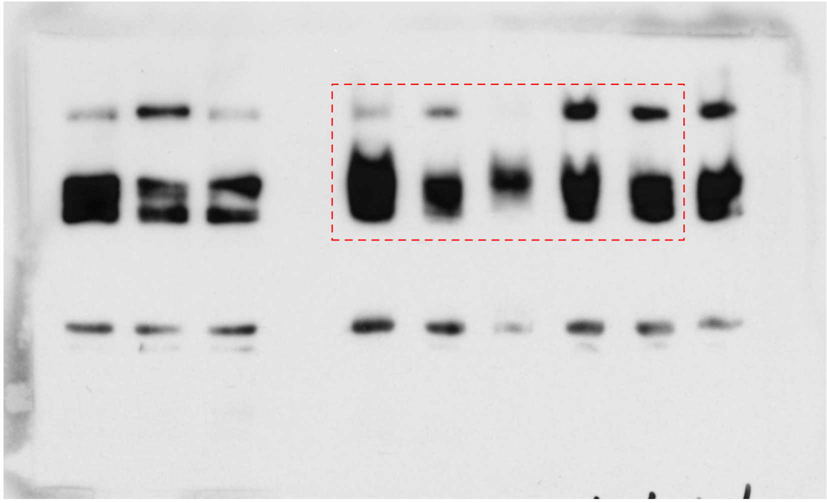

Actin

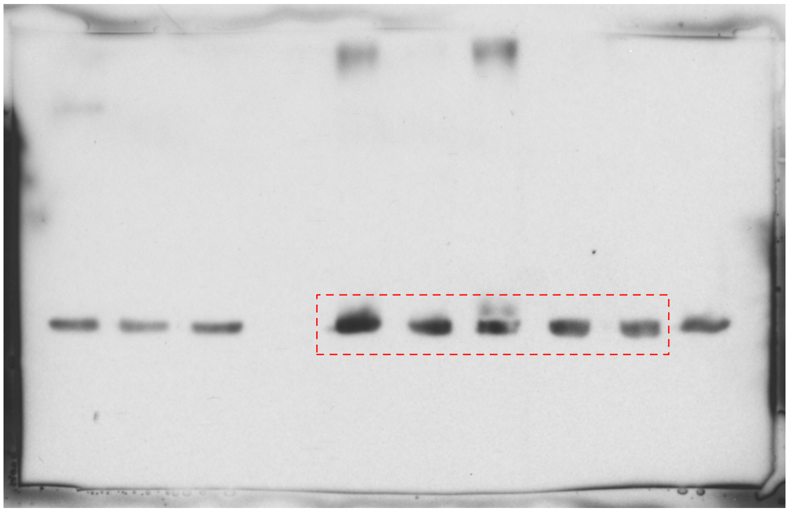

Figure 4 B

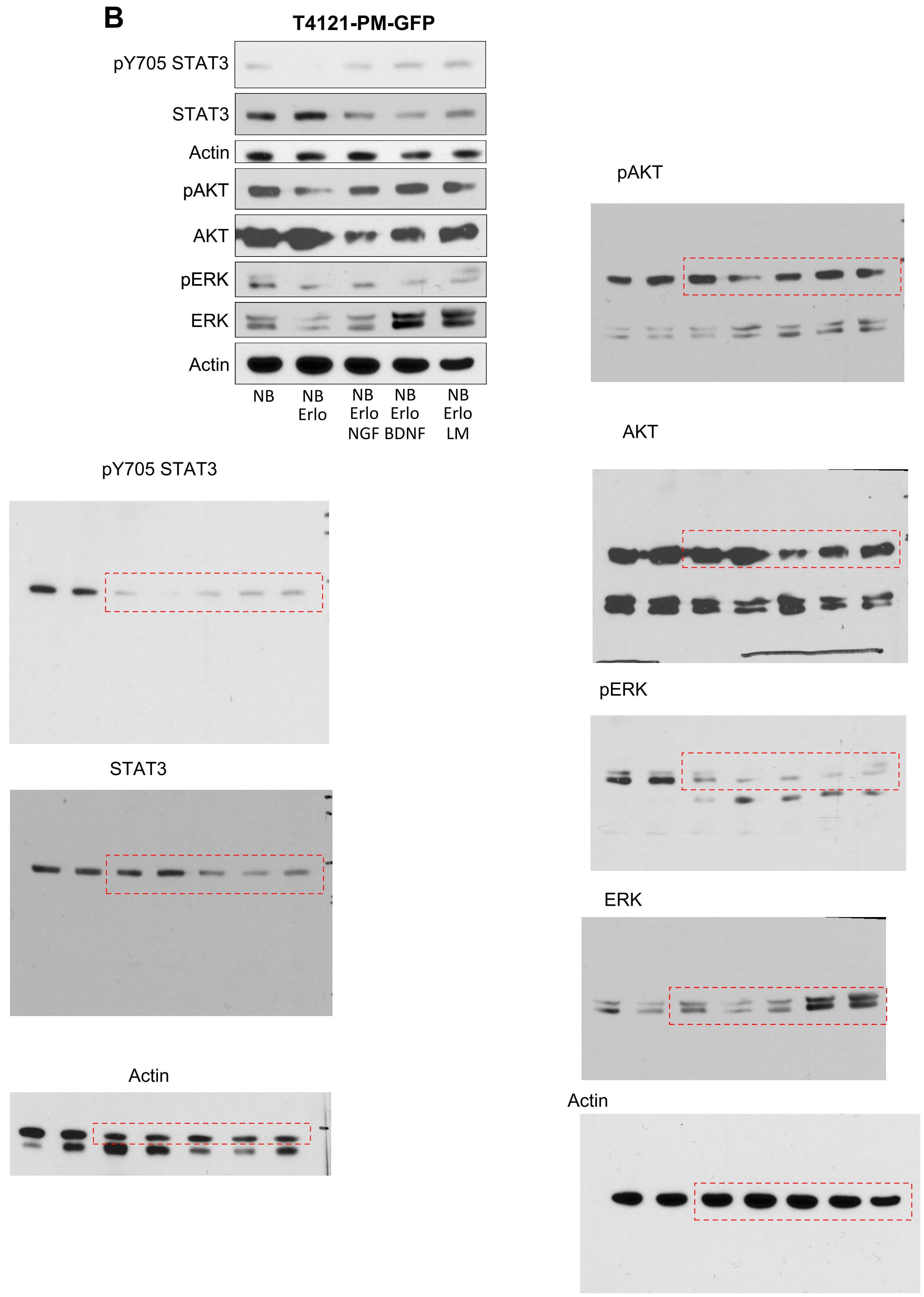
B
T4121-PM-GFP
pY705 STAT3
STAT3
Actin
pAKT
AKT
pERK
ERK
Actin
NB
NB Erlo
NB Erlo NGF
NB Erlo BDNF
NB Erlo LM
pAKT
AKT
pY705 STAT3
pERK
STAT3
ERK
Actin
Actin

Figure 4C

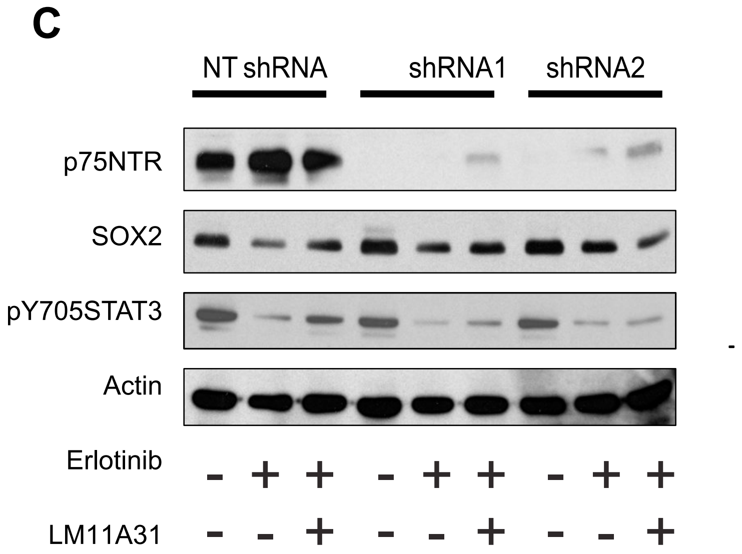

p75NTR

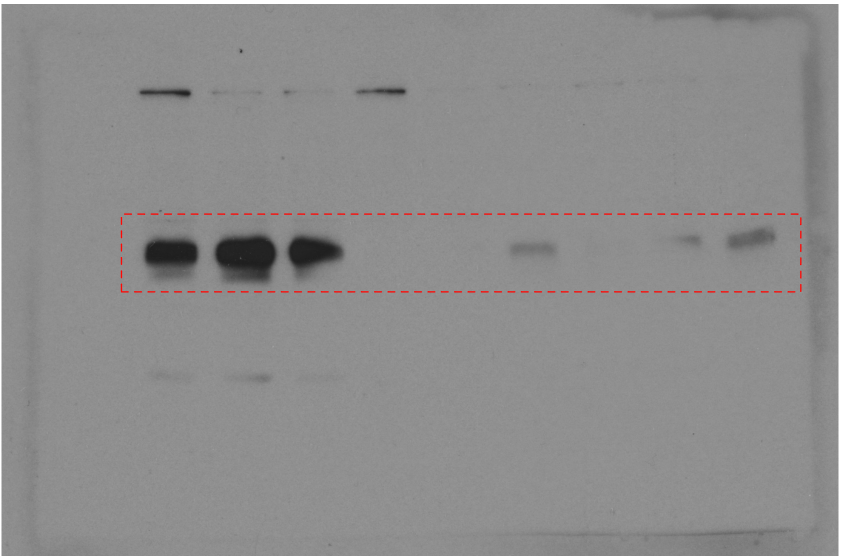

pY705STAT3

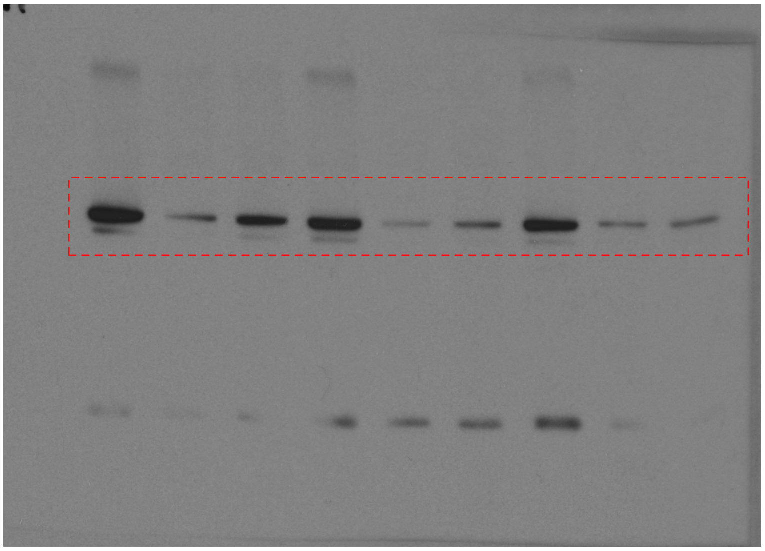

SOX2

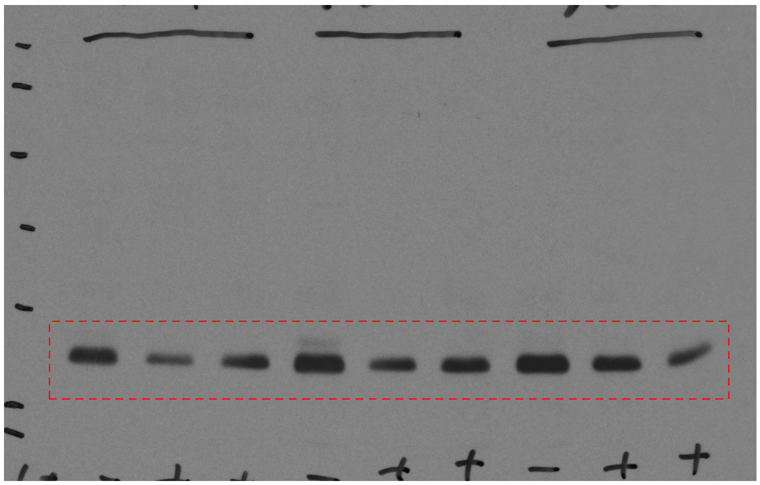

Actin

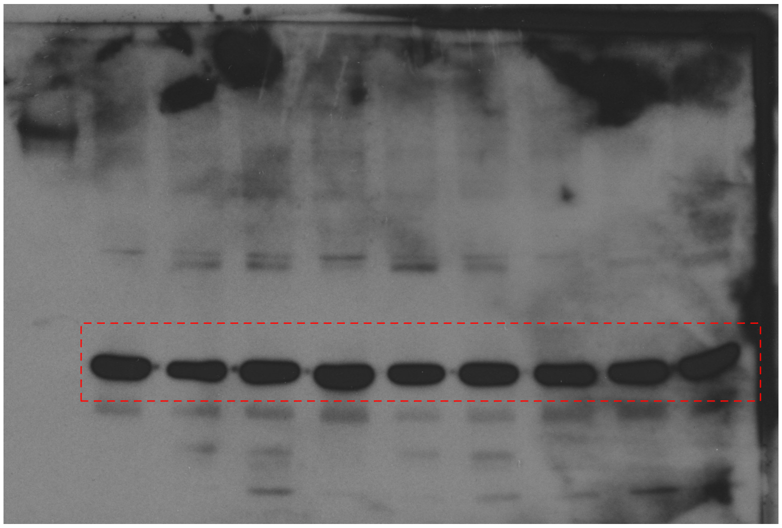

Supplemental Figure 3A

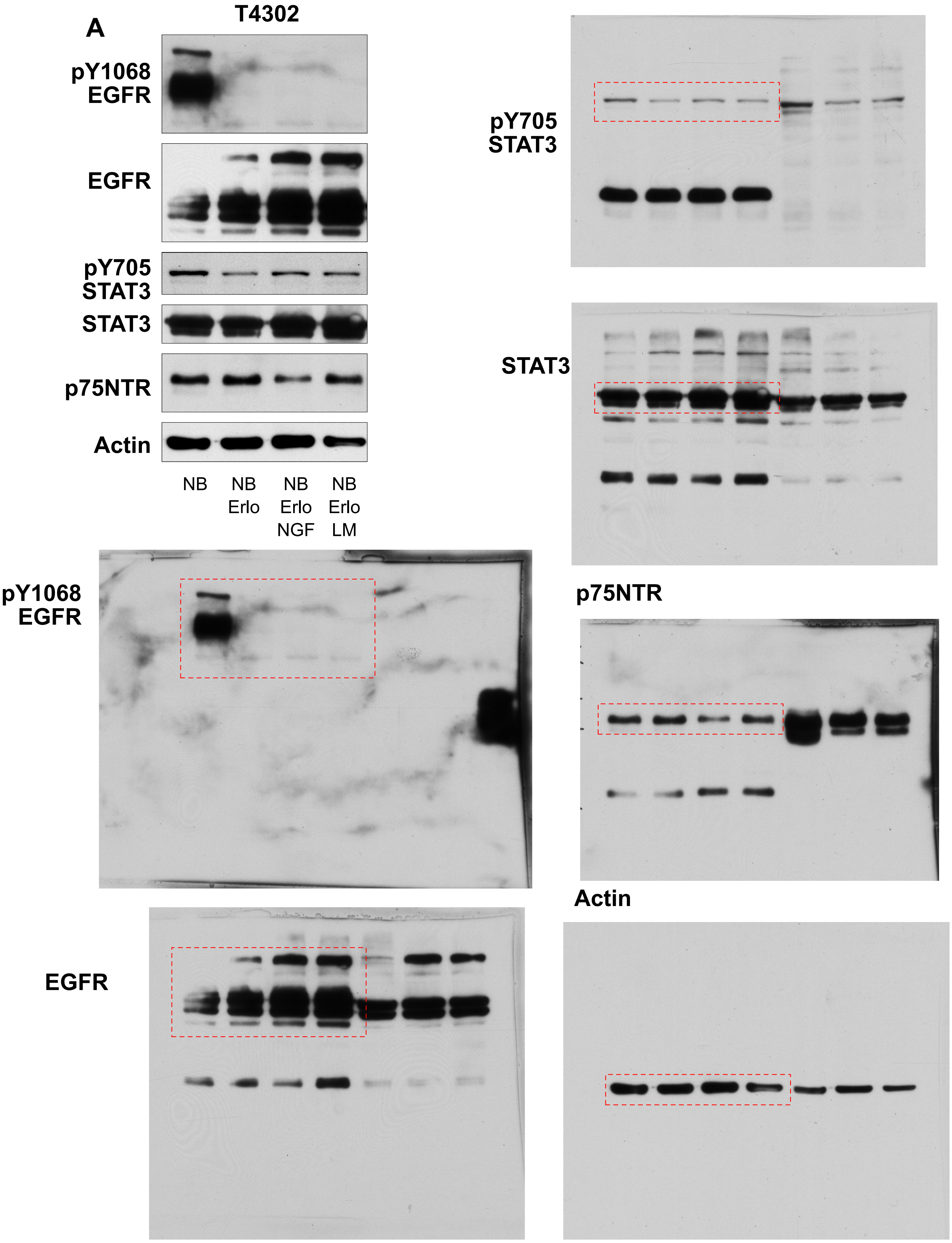

Supplemental Table 1 RESOURCES

REAGENT or RESOURCE	SOURCE	IDENTIFIER
Antibodies and detection systems		
CD133/1	Miltenyi Biotec	Cat# 130-092-395, RRID:AB_615061
EGFR	Abcam	Cat# ab32077, RRID:AB_732101
EGFR	Ventana Medical Systems	Cat# 790-2988
p75NTR	Millipore	Cat#05-446, RRID: AB_309737
p75NTR	Cell Signaling Technology	Cat# 8238, RRID:AB_10839265
P75NTR	Millipore/Cell Marque	Cat# 304M-14
phospho-STAT3 Tyr705	Cell Signaling Technology	Cat# 9145, RRID:AB_2491009
STAT3	Cell Signaling Technology	Cat# 4904, RRID:AB_331269
phospho-AKT Thr450	Cell Signaling Technology	Cat# 12178, RRID:AB_2734744
AKT (pan)	Cell Signaling Technology	Cat# 4691, RRID:AB_915783
Phospho-p44/42 MAPK (Erk1) (Tyr204)/(Erk2) (Tyr187)	Cell Signaling Technology	Cat#5726
p44/42 MAPK (Erk1/2)	Cell Signaling Technology	Cat# 9102, RRID:AB_330744
phospho-EGFR (Tyr1068)	Cell Signaling Technology	Cat# 4031, RRID:AB_1903957
SOX2	R&D Systems	Cat# MAB2018, RRID:AB_358009
beta-Actin	Santa Cruz Biotechnology	Cat# sc-130301, RRID:AB_2223360
Donkey Anti-Rabbit IgG, DyLight 649 Conjugated	Jackson ImmunoResearch Labs	Cat# 711-495-152, RRID:AB_2315775
Cy3-AffiniPure Donkey Anti-Mouse IgG	Jackson ImmunoResearch Labs	Cat# 715-165-151, RRID:AB_2315777
OptiView DAB IHC Detection Kit	Ventana Medical Systems	Cat# 760-700
EnVision FLEX DAB+ Substrate Chromogen System	Dako/Agilent	Cat# GV825
Chemicals, Peptides, and Recombinant Proteins		
Geltrex	Life Technologies	Cat# A1413201
LM11A-31 dichloride	Sigma-Aldrich	Cat# SML0664
NGF-beta human recombinant	R&D Systems	Cat# 256-GF

BDNF human recombinant	STEMCELL Technologies<br>Cell Signaling Technology<br>R&D Systems	Cat # 78005<br>Cat # 3897S<br>Cat # 248-BD
poly-lysin	Sigma-Aldrich	Cat # P8920
Erlotinib	Cayman Chemical Company	Cat# 10483
Temozolomide	Santa Cruz Biotechnology	Cat# 85622-93-1
Lipofectamine 2000	Life Technologies	Cat# 11668019
Biotool DNA transfection reagent	Biotool (biomake.com)	n/a
G418	Thermo Fisher Scientific	Cat # 11811023
thymidine	Sigma-Aldrich	Cat #T1895
human EGF recombinant	R&D Systems	Cat # 236-EG
human FGF recombinant	R&D Systems	Cat # 233-FB
Critical Commercial Assays		
CellTrace Far Red Cell Proliferation Kit	Thermo Fisher Scientific	Cat# C34564
Cell Titer Glo	Promega	Cat# G7572
Bio-Rad Protein Assay Dye	BIO-RAD	Cat# 5000006
ECL Plus Western Blotting Substrate	Pierce Biotechnology	Cat # 32132
Papain Dissociation Kit	Worthington Biochemical	Cat# LK003150
Experimental Models: Organisms/Strains		
Mouse: NOD.Cg-Prkdcscid Il2rgtm1Wjl/SzJ	The Jackson Laboratory	JAX: 005557
Recombinant DNA		
ps.PAX2	Addgene	Plasmid #12260
pMD2.G	Addgene	Plasmid #12259
pLKO.1 non-targeting vector	Sigma-Aldrich Mission	SHC002
p75NTR shRNA vector 1	Sigma-Aldrich Mission	TRCN0000058153
p75NTR shRNA vector 1	Sigma-Aldrich Mission	TRCN0000058156
PM-GFP	Addgene	Plasmid #21213

Software and Algorithms		
Extreme limiting-dilution analysis	Hu and Smyth, 2009	http://bioinf.wehi.edu.au/software/elda/
ImageJ	https://imagej.nih.gov/ij/	v1.52k
R	https://www.r-project.org/	R version 3.4.1
RStudio	https://www.rstudio.com/	Version 1.0.136
KaleidaGraph	Synergy Software	Version 4.1.3
HASCIA	Chumakova et al., 2019	https://hascia.lerner.ccf.org/
